# A lightweight deep learning framework for fast, real-time super-resolution fluctuation imaging

**DOI:** 10.1016/j.bpr.2026.100279

**Published:** 2026-08-06

**Authors:** Miyase Tekpınar, Jelle Komen, Hana Valenta, Ran Huo, Klarinda de Zwaan, Peter Dedecker, Nergis Tomen, Kristin Grußmayer

**Affiliations:** 1 Department of Bionanoscience and Kavli Institute of Nanoscience, TU Delft, Delft, the Netherlands; 2 Department of Intelligent Systems, TU Delft, Delft, the Netherlands; 3 Department of Chemistry, KU Leuven, Leuven, Belgium; 4 Biomedical Engineering, Nanoscopy for Nanomedicine, TU Eindhoven, Eindhoven, the Netherlands

**Keywords:** real-time super-resolution imaging, low latency, AI, convolutional gated recurrent unit, convGRU, fluctuation microscopy, super-resolution optical fluctuation imaging, high-throughput imaging

## Abstract

Capturing dynamic cellular processes in live cells requires fast imaging with very high resolution beyond the diffraction limit. Fluctuation-based super-resolution techniques overcome this limit by exploiting correlations in fluorescence blinking, but they typically require hundreds of frames and computationally intensive post-processing, prohibiting real-time imaging of fast cellular events. Recent deep learning approaches aim to increase temporal resolution; however, many rely on extensive pre-processing or large, complex models that increase training costs and inference latency, preventing real-time deployment. To address this, we employ a lightweight recurrent neural network model that integrates sequential low-resolution frames to extract spatio-temporally correlated signals. It significantly improves temporal resolution by reducing the required number of frames down to as few as 8 frames while doubling the spatial resolution in an inference time under 30 ms. Furthermore, gentle imaging conditions are essential for extracting reliable biologically relevant information, especially in long-term experiments. Our method is suitable for live-cell imaging under extreme signal/noise ratio conditions, allowing imaging under very low laser intensities to prevent photodamage. By combining simulation-based training with an efficient network architecture, we introduce real-time super-resolution fluctuation imaging (RESURF), a deep-learning-based real-time super-resolution fluctuation imaging framework. We demonstrate that RESURF generalizes across different biological structures and can be readily adapted to various microscope setups using a small data set for transfer learning. The accompanying data set, comprising simulations and experiments across multiple subcellular structures and labeling strategies, establishes a benchmarking platform for fluctuation-based super-resolution techniques. RESURF offers a practical, low-latency deep-learning framework for high-throughput imaging and real-time, smart live-cell super-resolution imaging.

## Why it matters

Studying fast processes in living cells requires imaging methods that are both high resolution and fast. Traditional fluctuation-based super-resolution techniques can exceed the diffraction limit, but they often need hundreds of images and heavy computation, preventing real-time imaging. We present RESURF, a lightweight deep learning method that combines information from a sequence of low-resolution images. RESURF reduces the number of required frames to as few as 8 while doubling spatial resolution and producing results in under 30 ms. It also works under very low-light conditions, helping to avoid cell damage during long experiments. RESURF adapts easily to different microscopes and biological samples, enabling practical, real-time super-resolution imaging and providing a benchmark data set for future research.

## Introduction

Live-cell fluorescence imaging captures dynamic cellular behaviors and aims to maximize both spatial and temporal resolution while minimizing sample damage,^1^ enabling advancements in fundamental cell biology. However, visualizing these cellular dynamics is constrained by the diffraction limit, which restricts the spatial resolution to around 200–300 nm. Super-resolution (SR) techniques can overcome this barrier and achieve higher spatial resolutions.^2^^,^^3^^,^^4^ Single-molecule blinking-based SR techniques use the stochastic switch between a bright “on” state and a dark “off” state and can be performed with a wide-field (WF) microscope. Under sparse blinking conditions, single-molecule localization microscopy (SMLM) can be used to localize individual molecules and generate SR images requiring many thousands of frames, usually corresponding to data acquisition over minutes to hours. In addition to SMLM, structured illumination microscopy (SIM), stimulated emission depletion microscopy (STED), and the like use patterned illumination to modulate fluorescence behavior to reach SR. Although fast-imaging techniques are offered for both techniques, they require a complex microscopy setup.^5^^,^^6^ As an alternative, fluctuation-based SR techniques need only a WF microscope and hundreds of frames to reconstruct an SR image, and they can handle high densities of blinking fluorophores.^7^ Widely used examples of fluctuation-based methods are SR optical fluctuation imaging (SOFI)^7^^,^^8^^,^^9^ and SRRFs (SR radial fluctuations).^10^^,^^11^ In contrast to other SR techniques, SOFI leverages the correlation of blinking from a fluorophore over time and space and the independence of different fluorophores.^8^^,^^12^ Dertinger et al. showed that $n^{\text{th}}$-order cumulants of a diffraction-limited image sequence of blinking emitters result in a point spread function (PSF) raised to the power of n, which brings a $\sqrt{n}$-fold better resolution. This resolution enhancement can be further increased to *n*-fold in $n^{\text{th}}$-order SOFI by applying linearization and deconvolution or Fourier reweighting after the cumulant calculation.^13^ Another common technique is SRRF, which operates by computing gradient maps and applying a radiality transform that exploits the radial symmetry of the PSF. A final cumulant-based analysis of the image sequence produces a super-resolved, noise-suppressed image. The enhanced version, eSRRF, further assists users in selecting parameter settings that minimize the risk of artifacts.^11^ Even though the aforementioned methods require shorter imaging time than SMLM, their limited speed restricts applications to slow cellular dynamics. In addition, SR image reconstruction is performed after image acquisition (off-line), and the computation typically takes seconds to minutes.^14^ Therefore, these approaches are not suitable for real-time, simultaneous image acquisition and SR image reconstruction, desirable for high-throughput and smart microscopy and, in particular, for live-cell imaging. These challenges have motivated the development of computational strategies aimed at enabling faster^12^ and more efficient reconstruction.^15^^,^^16^

Deep learning has been extensively explored to increase image quality and imaging speed.^17^ Most methods employ the U-Net architecture,^18^ a type of convolutional neural network (CNN), owing to its high performance in image segmentation, denoising, and deconvolution.^19^^,^^20^^,^^21^^,^^22^^,^^23^ U-Net is also used to accelerate SRRF imaging.^23^ However, it restricts input image size and requires an interpolation step prior to U-Net inference, limiting its real-time deployment. In addition to CNNs, generative adversarial networks (GANs) can, in principle, be used for SR image generation. Single-frame deep-learning super-resolution microscopy (SFSRM) employs a GAN architecture called enhanced super-resolution generative adversarial network (ESRGAN) and a single frame with an edge map as the network input.^24^ In addition to edge-map analysis, this method also requires a denoising step for low-single/noise ratio (SNR) conditions prior to ESRGAN, and the large size of the model results in a high computational latency of hundreds of ms of inference time, preventing real-time application.

Despite the growing number of studies that apply deep learning in microscopy, few AI-based methods focus on exploiting temporal correlations.^25^^,^^26^ Recurrent neural networks (RNNs) are a notable deep learning architecture to leverage temporal information and are rarely used in microscopy. An example of an RNN-based SR technique, specifically a long-short-term memory (LSTM) network, is DBlink for video reconstruction from SMLM data.^26^ By feeding localization maps into the LSTM, DBlink generates SR videos at rates of up to 66 frames per second. However, this method remains constrained to SMLM sample conditions and relies on localization-based processing, restricting its use to off-line image analysis.

In this study, we offer a deep learning framework to achieve real-time super-resolution fluctuation (RESURF) from few, ultra-low-SNR microscopy images. RESURF enables real-time deployment by both reducing imaging time and allowing a pre-processing-free, fast inference time with only raw microscopy images as input. The reduced computational complexity and efficient training also result in reduced power consumption, which is crucial given the growing use of AI in science and medicine.

We chose a deep learning architecture based on a lightweight RNN architecture capable of leveraging both temporal and spatial information, called multi-image SR gated recurrent unit (MISRGRU^27^). Originally designed to predict single SR images from multiple low-resolution images in remote sensing, MISRGRU utilizes a convolutional gated recurrent unit (convGRU) to process sequential information. This allows the network to learn the spatio-temporal correlations between frames via blinking emitters and makes it compatible with any fluctuation-based SR technique. As a target output, we use second-order SOFI images to achieve a 2-fold resolution improvement while limiting the computational demand of data preparation for training. We show that it can be applied to bioimaging, which, in contrast, operates under low-light conditions, delivering high-numerical-aperture, diffraction-limited microscopy images. MISRGRU is flexible in that it supports training and inference with an arbitrary image size. By design, the output can be easily interpolated to any image size, allowing for straightforward training to produce *n*-fold larger output images and facilitating *n*-fold resolution improvement.

We used large synthetic data to create multiple foundation models, where we have full access to ground-truth (GT) structures for evaluation. Our simulated data set features microtubule-like structures with different SNR levels, dynamic out-of-focus background, and a realistic sCMOS camera noise model. We tested different input frame counts for the network to determine the model performance and the temporal resolution. Compared to the hundred or more frames typically required for second-order SOFI, our models achieved a 2-fold spatial resolution improvement for very low-SNR conditions using as few as 8 input frames. To allow real-time deployment, we optimized the size of the network and achieved an inference time of less than 30 ms. We also showed the generalization of the model to different patterns using both experimental and simulated data. Furthermore, by sharing our simulations and experimental data set, including multiple subcellular structures and labeling strategies, we offer our community a benchmarking platform for fluctuation-based microscopy methods.

Next, we demonstrate that we can optimize foundation models via transfer learning with a small data set for a range of live-cell and fixed-cell data labeled with different blinking fluorescent proteins and organic dyes. Using realistic simulations with parameters close to experimental data for retraining, the network generalized to different structures and outperformed SOFI and eSRRF. In addition, we demonstrate that foundation models can learn different microscopy configurations from limited experimental data. This allows users to have flexibility in training strategies that can be conveniently adapted to facilitate dynamic and high-throughput SR experiments.

## Materials and methods

### Simulations

The MATLAB-based SOFI simulation tool was adapted to more accurately represent noise and background effects, following the methodology described in Girsault et al.^28^ To account for electronic noise, a gain and standard deviation map of the sCMOS camera used in our experimental setup was generated and incorporated into the simulation.^29^^,^^30^ In addition, a nonstationary background model was implemented in which background signals arise from out-of-focus blinking emitters. Inspired by the approach used in super-resolution imaging based on autocorrelation with two-step deconvolution (SACD),^12^ the distribution of emitters in the focal plane was geometrically shifted and transformed to generate a corresponding background distribution, which was subsequently convolved with a broader PSF to simulate out-of-focus blur. For the simulations prepared for live samples, we added random emitters in the focal plane and in some random axial planes outside the focal plane.

The simulation begins by generating a binary mask of the target structure, which consists only of filamentous patterns for training data sets of all the models presented in this study. We also created hollow mitochondrial structures to test the generalization of the model to different structures. These patterns are produced by modifying the pattern generation algorithms originally developed for the DBlink method and to study mitochondrial dysfunction.^26^^,^^31^ Emitters were randomly distributed across the simulation area at a density of $5,000\;\text{fluorophores}/\mu m^{2}$, and only those overlapping with the binary mask were retained. The spatial grid for emitter placement was set to 1,280 × 1,280 pixels with a resolution of 10 nm per pixel. Filament structures with thicknesses ranging from 10 to 40 nm were simulated.

The temporal blinking behavior of individual emitters was simulated using a continuous-time Markov process,^28^ resulting in high-resolution video sequences. The emitters have an average on time of 10 ms and averaged off times of 600, 1,200, or 2,400 ms to model different blinking speeds. The SNR was further controlled by adjusting the ion value, which represents the average number of photons emitted per frame by each fluorophore. Ion values of 100, 80, 60, and 40 were used to reflect different SNR conditions, mimicking the photophysics of fluorescent proteins under low illumination.

Both in-focus and out-of-focus emitter planes were convolved with Gaussian PSFs, with full-width at half-maximum (FWHM) values of 220 and 2,000 nm, respectively. The maximum background contribution per pixel was calculated based on the mean brightness of fluorophores in the focal plane, using a fixed ratio between in-focus and out-of-focus signals. The resulting noise-free image stack was then downsampled by a factor of 10. Finally, Poisson-distributed photon noise and sCMOS-specific electronic noise were added to each frame to produce the final simulated data set.

These parameters are changed for fluorescent protein simulations to represent higher background and low-SNR conditions, as shown in Figures S1 and S2. The on time and off time are adjusted for the fluorescent proteins, specifically for ffDronpa.^32^ As a result, the on-time ratio happens to be faster than the simulations for the foundation models, which could decrease the frequency of the appearance of random single emitters. Therefore, we lowered the emitter density to $1,000\;\text{fluorophores}/\mu m^{2}$. As we used the foundation model to initialize the training, we only simulated 200 different microtubule patterns. Then, we varied the SNR and background levels by generating 10 different images for each pattern. Unlike previous simulations, we also added random emitters in the background, both in the focal plane and in different axial positions out of focus. In each 10-mer of simulation, we only added one high-SNR condition, and we maintained low-SNR and high-background conditions for the rest.

### Network and training scheme

**Figure 1:**
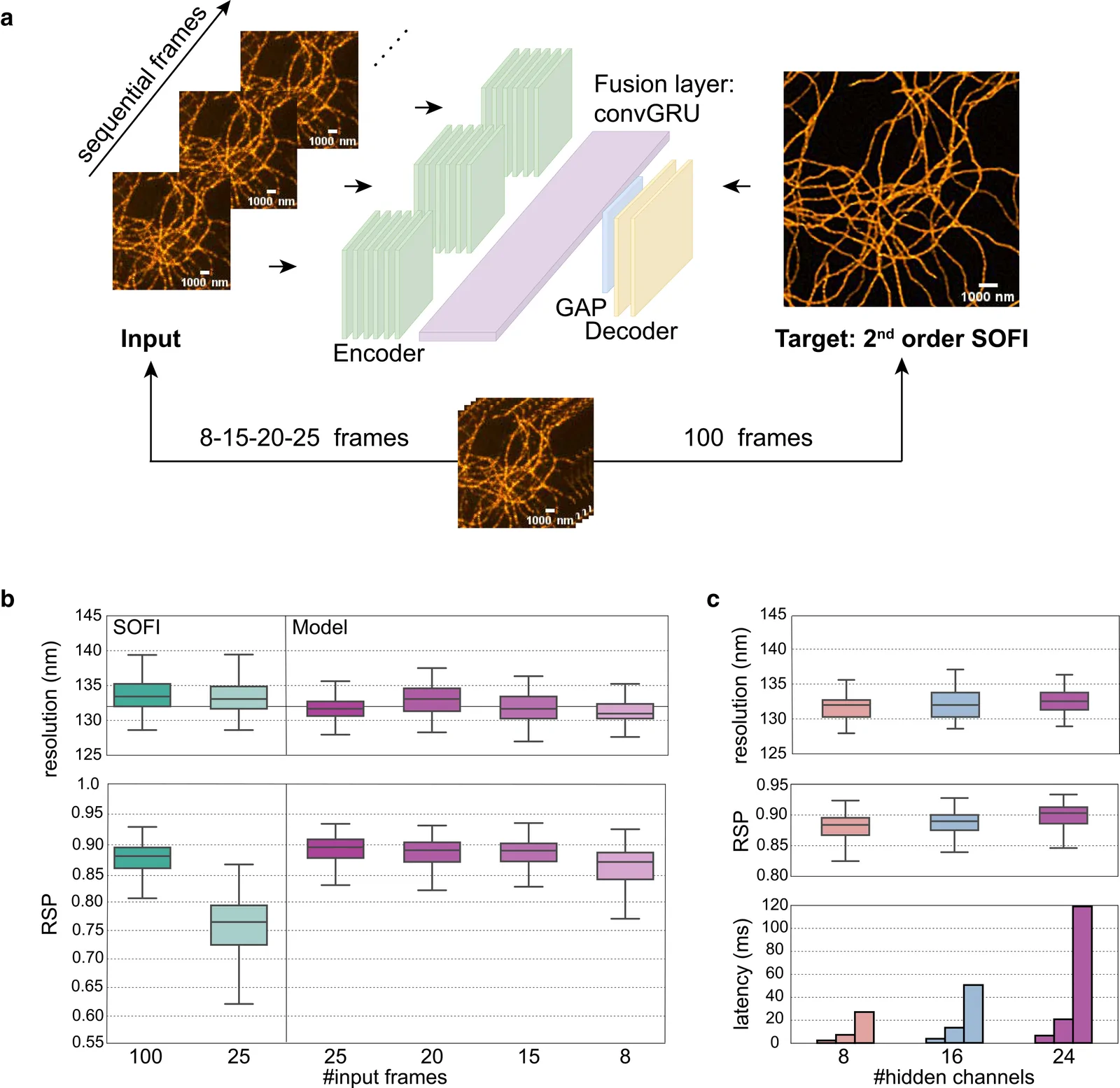
The network architecture and optimization for RESURF. (a) The network architecture (MISRGRU) and training strategy. It is trained with simulated diffraction-limited image sequences and corresponding second-order SOFI reconstructions. Multiple models (foundation models) are trained by using different numbers of input frames (8, 15, 20, and 25) and different numbers of hidden channels. (b) Spatial resolution estimated via image decorrelation analysis and resolution-scaled Pearson correlation coefficient for SOFI (green) and the models (purple) using different numbers of input frames. Each boxplot depicts the median value (center line), 25th and 75th percentiles (box edges), and 1.5 IQR (interquartile range) (whiskers). The line at 132 nm marks the half-resolution of the wide-field average image. (c) Performance evaluation of models configured with different numbers of hidden channels: 8 (red), 16 (blue), and 24 (purple), all using 20 frames as input. Latency is the average inference time over 500 repetitions (with TensorRT optimization): from left to right, each bar represents a different field-of-view (FOV) size: 128 × 128, 256 × 256, and 512 × 512 pixels. All models shown in this figure are trained using Fourier (F) loss. The comparison of different loss functions can be found in Figure S1. Scale bar: 1μm.

Multi-image SR (MISR) methods have been extensively studied in satellite imaging.^33^ MISRGRU was first introduced in 2020 to the computer vision community to improve the quality of satellite imagery.^27^ This model leverages multiple low-resolution images and an encoder-decoder framework to generate a super-resolved output. As illustrated in Figure 1, the architecture comprises an encoder, a convGRU serving as a fusion layer, and a decoder. We inherited this architecture and modified it to reduce the size and thus improve the latency by excluding the image registration layer and reducing the number of input channels from 64 to either 24 or 8. We also optimized the loss function by testing the mean absolute error as a spatial loss $\left(L_{1}\right)$ and a Fourier loss $\left(L_{F}\right)$.^34^ During training on live samples labeled by fluorescent proteins, we combined these two loss functions to optimize structural integrity and resolution of predictions. We updated the weights for spatial and Fourier losses every 20 epochs using gradient normalization.^35^

(1) $$L_{1}=\left|{\hat{Y}}_{u,v}-Y_{u,v}\right|$$

(2) $$L_{F}=L_{F_{A}}+L_{F_{∠}}$$

(3) $$L_{F_{A}}=\frac{2}{UV}\sum_{u=0}^{U/2-1}\sum_{v=0}^{V-1}|{\left|\hat{Y}\right|}_{u,v}-{\left|Y\right|}_{u,v}|$$

(4) $$L_{F_{∠}}=\frac{2}{UV}\sum_{u=0}^{U/2-1}\sum_{v=0}^{V-1}\left|∠{\hat{Y}}_{u,v}-∠Y_{u,v}\right|$$

Here, both SR images $\hat{Y}$ and the target image Y are transformed into the Fourier space by applying fast Fourier transform (FFT), where the absolute amplitude difference $L_{F_{A}}$ and absolute phase difference $L_{F_{∠}}$ are calculated. Due to symmetry in the Fourier space (Hermitian symmetry), only half of the spectral components are considered.

We trained the foundation model with simulated data sets of 128 × 128-pixel images. The data set was split into training (2,000 samples), evaluation, and test sets (480 samples each). Model training was performed using the Adam optimizer with a learning rate (η) of 0.001 and momentum parameters $\beta_{1}=0.9$ and $\beta_{2}=0.999$. Normal initialization was used for the model weights.^36^ To mitigate overfitting, early stopping was applied with a maximum of 400 training epochs and a patience threshold of 10. A batch size of 10 was selected to balance GPU memory usage and computational efficiency. All training and testing were conducted on an NVIDIA GeForce RTX 3090 GPU with 24 GB of memory.

To extend the model to experimental data, we used the pre-trained weights from the foundation model as initialization of the models we trained for live-cell and DNA-PAINT measurements. To further reduce inference latency, we applied TensorRT acceleration to all models trained within the PyTorch framework.^37^ This optimization included techniques such as layer fusion and precision calibration, including the use of lower-precision formats such as fixed-point floating points, to maximize computational efficiency.

### Performance evaluation

We evaluate the performance of SOFI reconstructions and AI-model-generated predictions using three complementary methods. First, we estimate image resolution through image decorrelation analysis, a technique that operates on a single image and relies on partial phase correlation to determine spatial resolution.^38^ Second, we employ the resolution-scaled Pearson correlation (RSP) metric to quantify the similarity between the SR image and a reference image.^39^ Prior to correlation computation, the SR image is adjusted to match the resolution of the reference image using an estimated resolution-scaling function (RSF), providing a more robust comparison than traditional metrics such as the structural similarity index (SSIM). For experimental data, the reference is the average projection of the image sequence (WF). As we noticed that the RSP score can underrepresent structural similarity in images with a high background, we also used a wavelet-based background and noise subtraction (WBNS) method to create a background-free alternative reference image.^40^ We used 3 levels in wavelet decomposition to estimate the background and skipped the denoising part of the algorithm. In addition, for the simulated test set, we also calculated the RSP scores between the GT and SR predictions. Lastly, to visualize the pixel-level difference between the reference image and the SR prediction, we used a resolution-scaled error (RSE) map. Similar to RSP, the absolute difference between pixel values is calculated after matching the resolution between two images and averaged to calculate the RSE score. We noticed that the boundary pixels in model predictions for actin data (in Figure 4d) sometimes show hot pixels. This is likely due to the extremely high signal levels in actin experiments, which are not represented in our training set. Therefore, we cropped the five boundary pixels in actin images and used the smaller cropped region for model evaluation with RSP and resolution analysis.

### Data analysis

#### SOFI

We reconstructed SOFI images using sofipackage based on a cross-cumulant-based algorithm, which is available at https://github.com/kgrussmayer/sofipackage. Following *n*^th^-order cross-cumulant analysis, images reach a resolution improvement of $\sqrt{n}$-fold. Next, we apply linearization and Richardson-Lucy deconvolution (RLD) as post-processing.^13^^,^^41^^,^^42^ To compensate for the nonlinearity in brightness, we use either linear scaling or adaptive linearization. While linear scaling takes the *n*^th^ root of the brightness in *n*^th^-order SOFI, adaptive linearization incorporates blinking properties to enable the effective use of the dynamic range. For RLD, we used a Gaussian kernel with the same FWHM we used for simulations and 10 iterations to reach a 2-fold resolution improvement. For foundation models, we use adaptive linearization and 2.2 pixels of FWHM for RLD. For high-background simulations, imitating live-cell conditions, we use linear scaling and 2.7 pixels of FWHM for RLD and follow with a wavelet-based background and noise subtraction (WBNS) method to eliminate the background and ringing artifacts caused by deconvolution.^40^ We used 3 levels of decomposition for background estimation and 2 levels of decomposition for noise estimation.

For synthetic training data sets created for live-cell experiments, we followed the WBNS method with a binary mask to eliminate the background pixels.^43^ To create the binary mask, we applied Li thresholding on the high-SNR versions in each 10-mer data set.

#### eSRRF

We used the Fiji plugin of eSRRF to obtain guidance on finding the best parameters for radius (R) and sensitivity level (S) and operated the rest of the analysis using the Python tool nanopyx.^10^^,^^44^ eSRRF has different types of temporal analysis. In all figures and evaluations, we used averaged eSRRF reconstructions with two-factor interpolation.

### Sample preparation and data acquisition

#### DNA-PAINT imaging

Fast DNA-PAINT data used in Figure 7 were acquired using a previously published method.^45^ Briefly, COS7 cells were cultured in DMEM with standard supplements, seeded on coverslips, and fixed. Microtubules in cells were stained first with primary antibodies against alpha-tubulin, followed by nanobodies conjugated with 5 × R1 DAN-PAINT docking strands (sequence 5′-TCCTCCTCCTCCTCCTCCT-3′). Cells were imaged in buffer consisting of 20 nM R1 imager strands (sequence 5′-AGGAGGA-3′, Atto655), 5% (v/v) ethylene carbonate, and 500 mM NaCl in PBS. A custom-built total internal reflection fluorescence (TIRF) microscope was used for imaging at 638 nm excitation $\left(2.5\;\text{kW}/cm^{2}\right)$; 10,000 frames were recorded at a 10 ms exposure using MicroManager. A full description of the nanobody production, labeling protocol, and microscope specifications is available in the method section of De Zwaan et al.^45^

#### Live-cell imaging

For live-cell imaging, HeLa cells were grown at 37°C in a 5% CO_2_ atmosphere in DMEM (Thermo Fisher Scientific) complemented with 10% fetal bovine serum (FBS) and GlutaMAX (Thermo Fisher Scientific). For imaging experiments, cells were seeded in 35-mm glass-bottom dishes (MatTek), and after 24 h, they were transiently transfected using FuGENE HD Transfection Reagent (Promega) according to the manufacturer’s protocol using the following plasmids: MAP4-ffDronpa (created in the Dedecker Lab), DAKAP-SkylanS (created in the Dedecker Lab), and SkylanS-betaActin (purchased from Addgene). 24 h after the transfection, the cells were washed with PBS (Thermo Fisher Scientific) and then imaged in PBS on a Nikon Eclipse Ti-2 inverted microscope equipped with a 1.4 NA oil immersion objective (100 CFI Apochromat TIRF) and a ZT405/488/561/640rpcv2 dichroic mirror with a ZET405/488/561/640 nm emission filter (both Chroma Technology). Imaging was always performed in TIRF mode using 488 and 405 nm lasers (Oxxius L6Cc laser combiner). Each measurement consisted of acquiring stacks of 500 or 1,000 images. MAP4-ffDronpa and DAKAP-SkylanS are recorded at a 30 ms exposure, and SkylanS-betaActin is recorded at a 20 ms exposure.

## Results

### Enabling ultrafast and real-time SR microscopy

SOFI typically requires several hundred frames (100–500), depending on fluorophore blinking characteristics and the SNR, to reach its theoretical 2-fold resolution enhancement and to produce high-SNR second-order SOFI reconstructions.^41^ However, this frame count and the associated computational time limit SOFI’s ability to achieve high temporal resolution for real-time SR sample screening. To address this limitation, we leverage deep learning to reduce the number of required input frames and computational time to enable real-time analysis while maintaining image quality comparable to high-SNR SOFI reconstructions.

The deep learning network architecture is based on the MISRGRU model, which comprises an encoder to extract feature representations of low-resolution frames, followed by a convolutional gated recurrent unit (convGRU)-based fusion layer that processes the features sequentially to capture correlations between input images. Global average pooling (GAP) combines them into a single representation, and a decoder reconstructs the high-resolution image by upsampling the fused feature maps. The input of the network consists of a time series of diffraction-limited frames, the target output being second-order SOFI reconstructions of the structures, as illustrated in Figure 1. To validate the network, we created a realistic synthetic data set by generating structural patterns resembling microtubules randomly decorated with fluorophores at a certain density. We simulated fluorophore blinking dynamics, including on and off states, and incorporated an sCMOS camera noise model along with a nonstationary background to closely mimic experimental imaging conditions (see materials and methods). The data set includes a range of SNR levels (photon number/frame: 40, 60, 80, and 100) and includes varying off times (600, 1,200, and 2,000 ms) while maintaining a constant average on time of 10 ms to mimic gentle, low-SNR live-cell conditions with fluorescent protein labels. To our knowledge, this is the first large public data set of fluctuation microscopy simulations intended to be used for training and benchmarking new methods. For generating SOFI target images, we used 100 diffraction-limited frames to compute second-order cross-cumulants, followed by linearization and RLD for post-processing.^13^ Following an approach similar to Chen et al., we trained separate models using 8, 15, 20, and 25 input frames to assess the network’s ability to reconstruct super-resolved images under different temporal constraints.^23^ We also tested both mean-absolute error (L1) and Fourier loss (F) and showed that Fourier loss performs better in reaching higher RSP scores for slow-blinking low-SNR conditions (Figure S1).

We determined the performance of the models with different input frames by applying a set of established evaluation metrics, as described in more detail in the performance evaluation part of the materials and methods section. We estimated the resolution of predicted SR images using decorrelation analysis, a widely adopted technique in SR microscopy for resolution quantification.^38^ We measured the similarity between the predicted and GT or WF images using the RSP coefficient and RSE, a standard method in SR imaging for quantitative comparison of image quality.^44^^,^^46^

In Figure 1b, we show the resolution and RSP scores for models with different numbers of inputs and compare them with the SOFI method. All models approximate the theoretical 2-fold improvement in spatial resolution of second-order SOFI. The RSP scores reveal that all models surpass SOFI in preserving structural integrity. While the Pearson correlation coefficient slightly decreases with fewer input frames, we find that the 8-frame model offers a favorable trade-off between temporal resolution and spatial accuracy for high-density samples. We optimized the network to limit computational latency and enable real-time deployment. We decreased the size of the model by systematically reducing the number of hidden channels while keeping the performance comparable to that of larger models. In Figure 1C, we show that the RSP and resolution scores change only slightly for the reduced model size, and the latency can be reduced to 27 ms for an image size of 512 × 512 pixels and 20 input frames. The 8-frame model can offer higher temporal resolution and computational speed with only 11 ms inference time. Despite the inherent complexity of sequential data processing in RNNs, our optimized MISRGRU models achieved a significantly lower end-to-end latency of less than 30 ms. This represents an approximate 400-fold acceleration in SR reconstruction compared to the SOFI method. These findings highlight the strong potential of MISRGRU for real-time, live-cell SR imaging. We further compare our deep learning framework with other approaches^23^^,^^47^ previously used for SR acceleration in Table 1. RESURF offers a lightweight, fast network model and a computationally manageable simulation-based training pipeline to enable accessible deployment for biological research.

**Table 1:** Comparison among different deep-learning-based super-resolution methods using different architectures

Method	Architecture	Input frames	Pre-processing	Training sample (size)	Parameters	Time (ms)
DL-eSRRF^23^	U-NET	5	interpolation	microtubules (1,005), CCPs (727)	31 M	66
SFSRM^47^	ESRGAN	1	edge map, denoising	synthetic random emitters (1,000), microtubules (300), vesicles (600)	33 M	732
RESURF	MISRGRU-hc24 MISRGRU-hc8	8, 20	not required	synthetic microtubules (2,000)	131 K, 15 K	65, 158 23, 57

Input frames are the required low-resolution images for the input of the network. DL-eSRRF uses eSRRF images and SFSRM uses a rendered localization microscopy image as a target. The Pre-processing column shows the required steps before the inference of the model. Training samples include each structure type with the number of image patches used during training. Parameters are the number of parameters to be trained for each model. Time shows inference latency of each model. It is benchmarked on the NVIDIA RTX A5000 GPU. For this table, we did not use any optimizations, such as TensorRT, to speed up inference.

**Figure 2:**
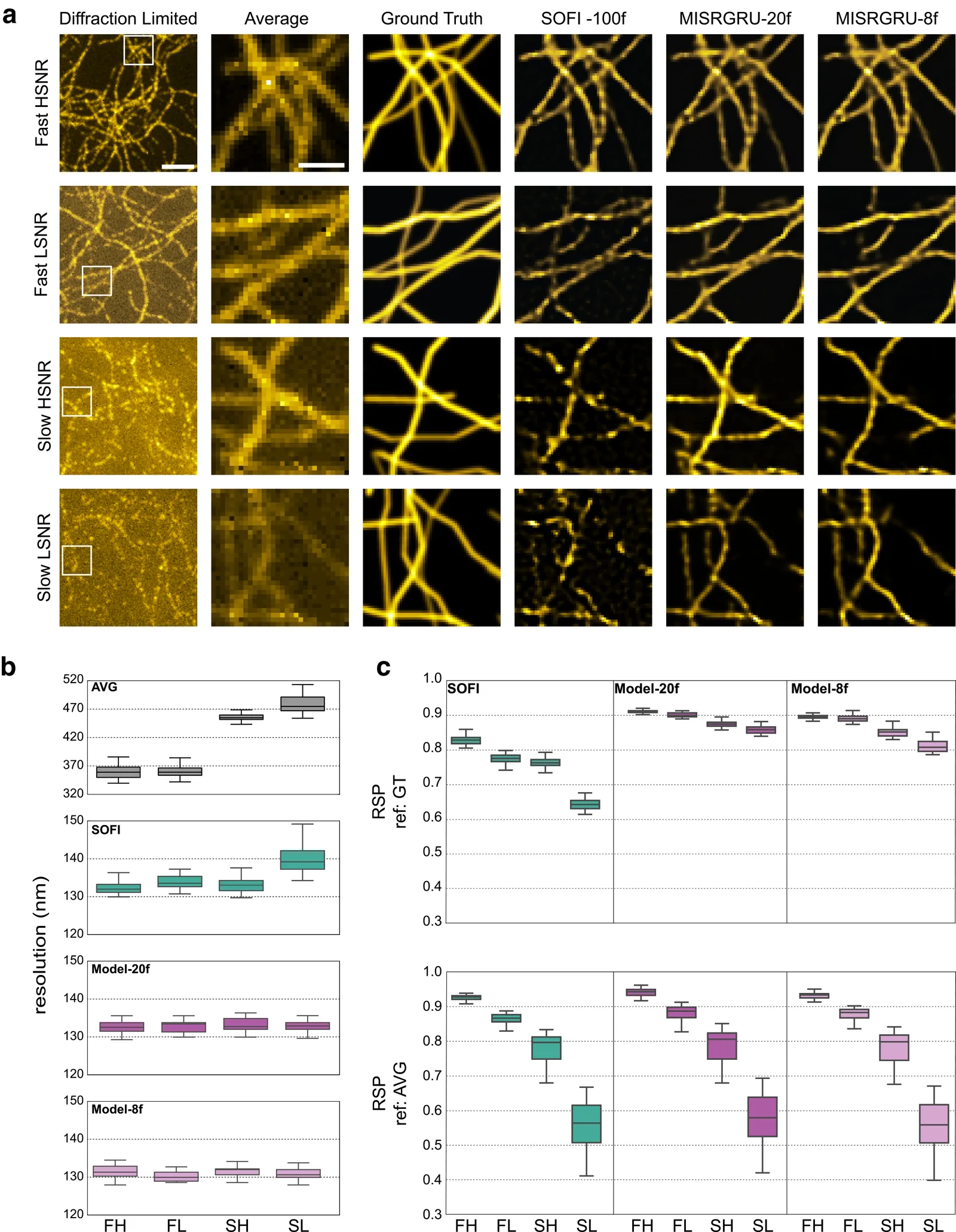
Performance of foundation models with simulations. (a) Qualitative comparison of models and SOFI. Each row corresponds to a different SNR (photons/frame): 100 (high SNR) or 40 (low SNR) and blinking conditions (on time/off time in ms): 10/600 (fast) or 10/2,400 (slow). From left to right: a single diffraction-limited input frame (128 × 128 pixels [px] or 12.8 × 12.8 μm), the average of the cropped area (30 × 30 px), the ground-truth (GT) image demonstrating 2-fold resolution enhancement, second-order SOFI reconstructions using 100 frames, and Model-20f and Model-8f. (b and c) Corresponding resolution estimations and RSP scores of averaged wide-field, SOFI, and the models for different conditions. RSP analysis is repeated for two different reference images: GT and averaged wide-field (AVG). FH, fast high SNR; FL, fast low SNR; SH, slow high SNR; SL, slow low SNR. Pixel size in AVG: 100 nm. Scale bars: 3μm (diffraction limited) and 1μm (average). Each boxplot depicts the median value (center line), 25th and 75th percentiles (box edges), and 1.5 IQR (interquartile range) (whiskers).

Visual examples of reconstructions at varying SNR levels and blinking conditions are provided in Figure 2. As expected, under the slowest blinking condition (Figure 2a), Model-8f misses some structures, simply because of the limited number of emitters switching to the on state in this short period of time. As we simulated the images with a Gaussian PSF and an FWHM of 220 nm, we first calculated the resolution of noise-free WF images (260 nm) and assigned half of this value (130 nm) to the 2-fold resolution improvement goal. However, image decorrelation analysis can be affected by the noise and background levels. Therefore, in Figure 2b, we see very high resolution values in WF images of slow-blinking conditions with worse SNRs and signal to background ratios (SBRs) compared to fast-blinking conditions. SOFI reconstructions using 100 frames fail to achieve a 2-fold resolution improvement under low-SNR and slow-blinking conditions. On the other hand, Model-20f and Model-8f maintain the resolution improvement under different conditions. This showed that by being trained on a simulation data set with varying SNR levels, the network learned to extract only the meaningful, correlated signal between subsequent frames, not the uncorrelated noise. In Figure 2c, we tested the RSP scores of SR reconstruction by taking both the GT and averaged WF frames as a reference in two separate analyses. The difference between the two analyses showed us that stationary background pixels in WF images degrade RSP scores if the SR reconstruction performs a good background elimination. WF images have very poor SNR levels under slow-blinking low-SNR conditions. As a result, RSP estimations using WF images resulted in lower scores than those using the GT as a reference. In the latter case, we can see the high performance of both models under extreme noise conditions (Figure 2c) compared to SOFI, more aligned with the images shown in Figure 2a. This offers a practical benefit for real-time super-resolution fluctuation (RESURF) live-cell imaging by tolerating lower laser intensities, thereby reducing phototoxicity and extending image acquisition durations.

In Figure S1, we show the performance of the network under SNR conditions in and out of range of the training set. Models achieve generalization to different, higher, and extremely low SNR levels that were not seen during training. Another model for higher SNR levels than the tested conditions can be easily retrained with a small data set (see transfer learning with small experimental data set). In Figure S5a, we evaluated the dependence of the model on the size of the training data set and showed that it converges to the minimum training error at around a training set size of 2,000. In addition, we tested the model’s performance for different combinations of training data sets (k-fold cross-validation analysis) and showed that models have high consistency, showing very similar scores in RSP and resolution analyses (Figure S5c). Furthermore, we tested the performance of the models for different image sizes and demonstrated that model performance does not degrade with increasing image size, even though it is only trained using images with a size of 128 × 128 pixels (Figure S6). This offers a widely applicable method for a large variety of typical imaging conditions using modern scientific cameras without additional effort to retrain the model.

### RESURF models generalize from simulations to different structures in live cells labeled by fluorescent proteins

Considering the dynamic features and poor SNR conditions of live-cell experiments, collecting a data set for training and processing the data to create SR target images can be very challenging. Therefore, we generated a new synthetic data set closely mimicking our experiments with different photoswitchable fluorescent proteins (ffDronpa and SkylanS) as explained in simulations. We used the specific, known parameters of the live-cell experiments to simulate their blinking kinetics and the FWHM for the Gaussian-shaped PSF obtained from our experimental data. A more detailed list of simulation parameters and network evaluation with simulations is provided in Figure S2. As the SNR and SBR levels of the simulations were extremely low, 100 frames were not enough to create high-SNR SOFI images as a target for training. To mitigate the risk that the network could learn background pixels containing deconvolution artifacts caused by SOFI analysis, we applied additional filtering on SOFI images by using wavelet-based background and noise subtraction (WBNS).^40^

**Figure 3:**
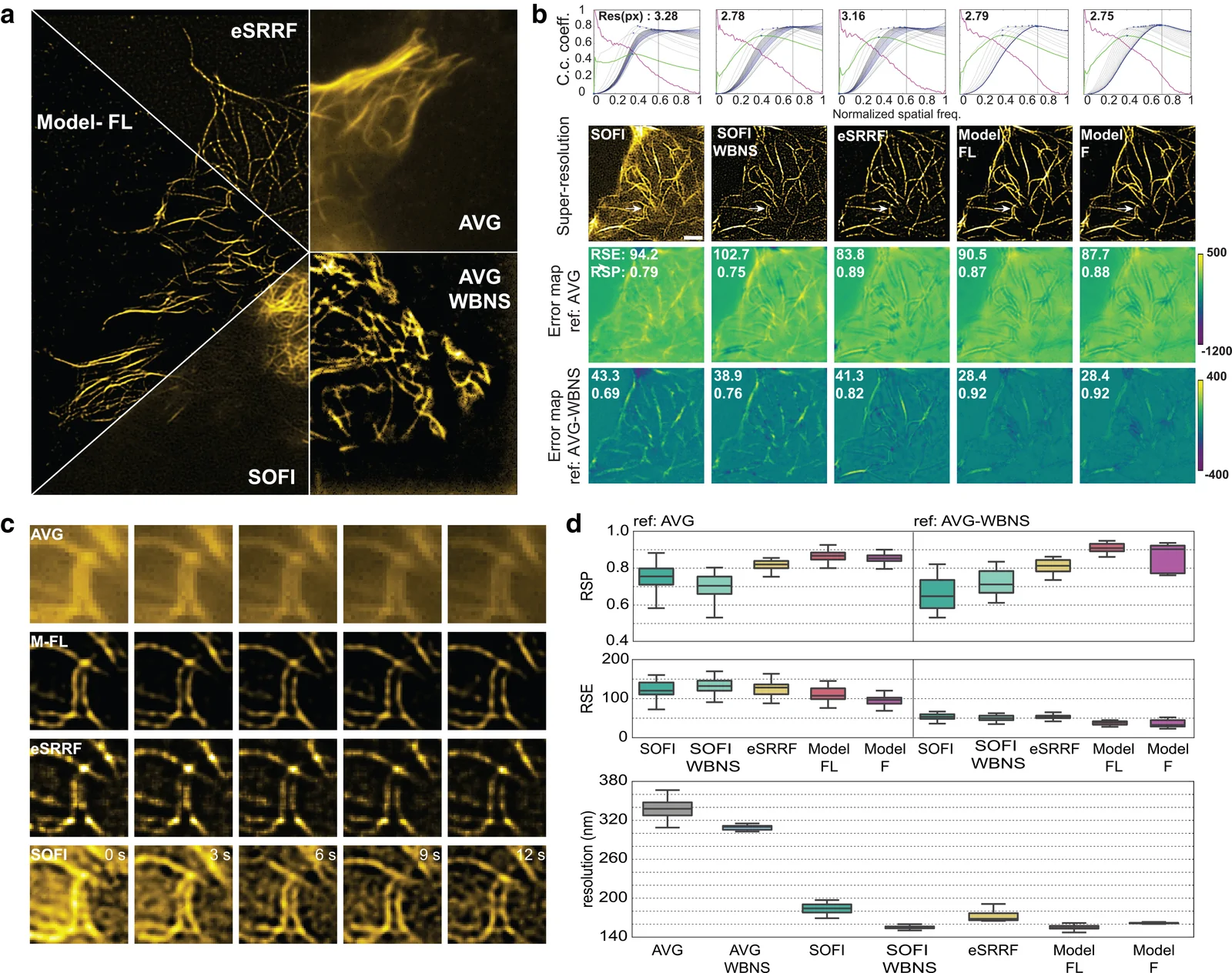
RESURF reconstructs SR images with high temporal resolution in a fast-bleaching live-sample. (a) Qualitative comparison of SR reconstruction of microtubule structures using Model-FL, SOFI, and eSRRF (HeLa cell and MAP4-ffDronpa). (b) Influence of background on image decorrelation analysis and resolution-scaled error (RSE) for cropped regions of 128 × 128 pixels. The first row shows decorrelation analysis for resolution estimation. Following SR reconstructions by different methods, RSE estimations for two reference images: (1) averaged projection of wide-field frames (AVG) and (2) background-subtracted AVG images using wavelet-based noise and background subtraction (AVG-WBNS). (c) SR reconstruction from a small dynamic region of 30 × 30 pixels (1.5 × 1.5 μm). (d) Evaluation scores for resolution estimation, RSP, and RSE using AVG and AVG-WBNS as reference images. Each boxplot is created by using 50 random crops of 128 × 128 from the large FOV in (a). Model-F, trained with Fourier loss; Model-FL, trained with a combination of Fourier and L1 loss. Pixel size in AVG: 108 nm. Scale bars: 1.6μm (b) and 2μm (c). Each boxplot depicts the median value (center line), 25th and 75th percentiles (box edges), and 1.5 IQR (interquartile range) (whiskers).

**Figure 4:**
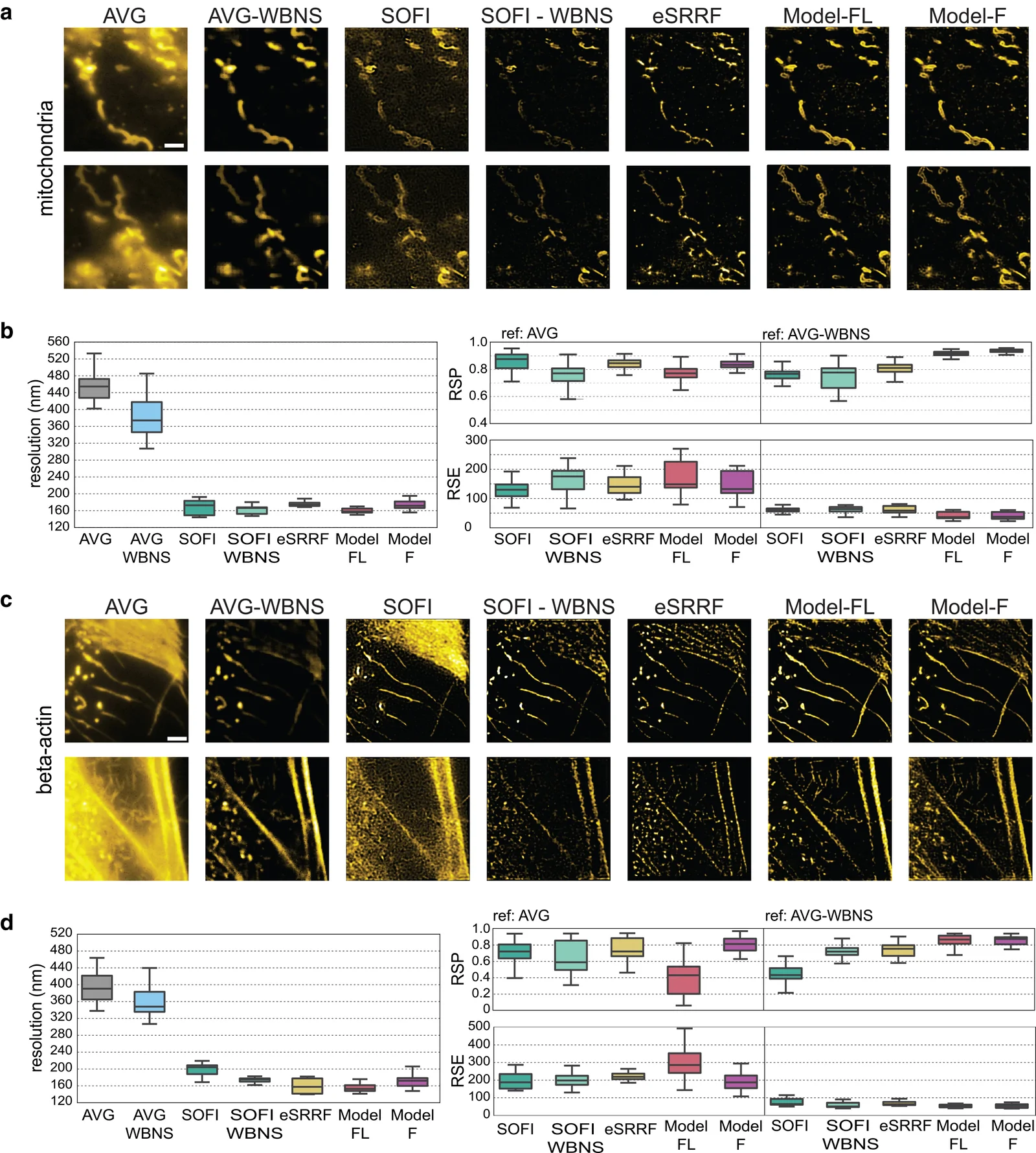
Generalization of RESURF to different intracellular structures. (a and b) Qualitative comparison of our models, SOFI and eSRRF, for a region of 128 × 128 pixels for mitochondria (DAKAP-SkylanS) (a) and actin (SkylanS-betaActin) in HeLa cells (c). (b and d) Corresponding evaluation scores for resolution estimation, RSP, and RSE using AVG and AVG-WBNS as reference images. Model-F, trained with Fourier loss; Model-FL, trained with a combination of Fourier and L1 loss. Pixel size: 108 nm. Scale bars: 2μm. Each boxplot depicts the median value (center line), 25th and 75th percentiles (box edges), and 1.5 IQR (interquartile range) (whiskers).

In Figures 3 and 4, we show the performance of RESURF in fast-bleaching live-cell measurements by testing it with different intracellular structures and different photoswitchable fluorescent proteins. As no GT image is available in this setting, we employed RSP to assess structural fidelity and RSE to show the difference between the reference image and the resolution-scaled version of the SR image.^46^ As previously mentioned for Figure 2, RSP analysis is sensitive to background pixels. Given that the majority of the field of view in most microscopy experiments will consist of background pixels, we repeated the RSP and RSE analyses using averaged WF (AVG) and post-processed WF (AVG-WBNS) as reference images. We compared the model results with SOFI images generated by 100 frames, SOFI images after post-processing with WBNS (SOFI-WBNS), and eSRRF images generated by 20 frames. Because of the higher background levels in this new data set, we additionally incorporated L1 loss together with Fourier loss (Model-FL) during training and compared it to the model using only Fourier loss (Model-F) (see section network and training scheme).

In Figure 3b, we evaluate the SR reconstructions by different methods. In the top row, we show the image decorrelation analysis graph for each SR image. The high background level in SOFI reconstruction influences image decorrelation analysis, resulting in a worse resolution estimation than SOFI-WBNS. eSRRF can eliminate background better than SOFI; however, it cannot reach the resolution level achieved by SOFI-WBNS and the models. Two very close microtubules that clearly demonstrate this difference are indicated by white arrows and are also shown in Figure 3c. Following the SR images, RSE maps for two different reference images are shown in the last two rows, together with the average RSE score and RSP coefficient. The RSP and pixel errors from RSE analysis are higher in the background when the reference image has a higher background, and the SR reconstruction eliminates the background. This effect can be clearly seen by comparing the RSE maps of SOFI and SOFI-WBNS for different reference images. Therefore, using AVG-WBNS as a reference gives more representative results for our models. The complete raw movie and SR reconstructions of the cell in Figure 3a are shown in Videos S1, S2, S3, S4, S5, and S6.

Video S1. Wide-field (TIRF) movie of 500 frames (20 fps), Figure 3

Video S2. Second-order SOFI (5 fps), Figure 3

Video S3. Second-order SOFI-WBNS (5 fps), Figure 3

Video S4. eSRRF(5 fps), Figure 3

Video S5. ModelFL(5 fps), Figure 3

Video S6. ModelF (5 fps), Figure 3

As shown in Figures 4a and 4c, our models trained only on microtubule structures generalize well to mitochondria and actin structures. This is confirmed by evaluation scores almost identical to microtubule structures. We also note that Model-F and Model-FL perform similarly: high-frequency spatial components such as microtubule and mitochondrial images need to be separated from low-frequency spatial components such as large cellular membranes or background signals. As Model-FL is trained with both Fourier and pixel-level loss function L1 while targeting background-subtracted SOFI images, it does not recognize the low-frequency spatial components (Figures 4c and 4d) and thus performs better in background elimination. Overall, both models achieved generalization to different structures and did not show any sign of memorizing the patterns.

For experiments with high-background conditions, eSRRF serves as a complementary benchmarking method. SOFI and eSRRF have distinct advantages and limitations. SOFI typically requires more frames and additional post-processing to perform well under high background levels, whereas eSRRF is known to achieve more effective background suppression with fewer frames. However, eSRRF can generate artifacts by collapsing closely spaced structures into one another.^48^ This effect is particularly evident in the mitochondrial images shown in Figure 4.

To achieve better generalization and avoid hallucinations, we followed several strategies. First, in addition to different SNR levels, we simulated different background levels and random emitters in different axial positions. Second, we optimized the SOFI targets to represent the information in the diffraction-limited input images. Lastly, we kept the emitter density levels moderate and the number of input frames low; thus, there are more random single emitters on the focal plane to avoid structural bias. In addition to successful models, we also show the counterexamples in Figure S3. When we trained the model to target the SOFI results using high SNRs and an order-of-magnitude more frames, the model showed more hallucinations in the background. Next, we trained the network by increasing the emitter density. The resulting model learned to connect random emitters to create filamentous structures, showing high structural bias.

In summary, we calibrated our training data set to have a large variety of SNR and background levels while avoiding complete structures in SOFI targets and eliminating background via post-processing when necessary. As a result of our training strategy and the sequential processing ability in the convGRU layer, the models learn to extract correlations between low-resolution images rather than recognizing patterns similar to the ones in the training data set.

### Applying RESURF models on public data sets with different fluorescent labels

**Figure 5:**
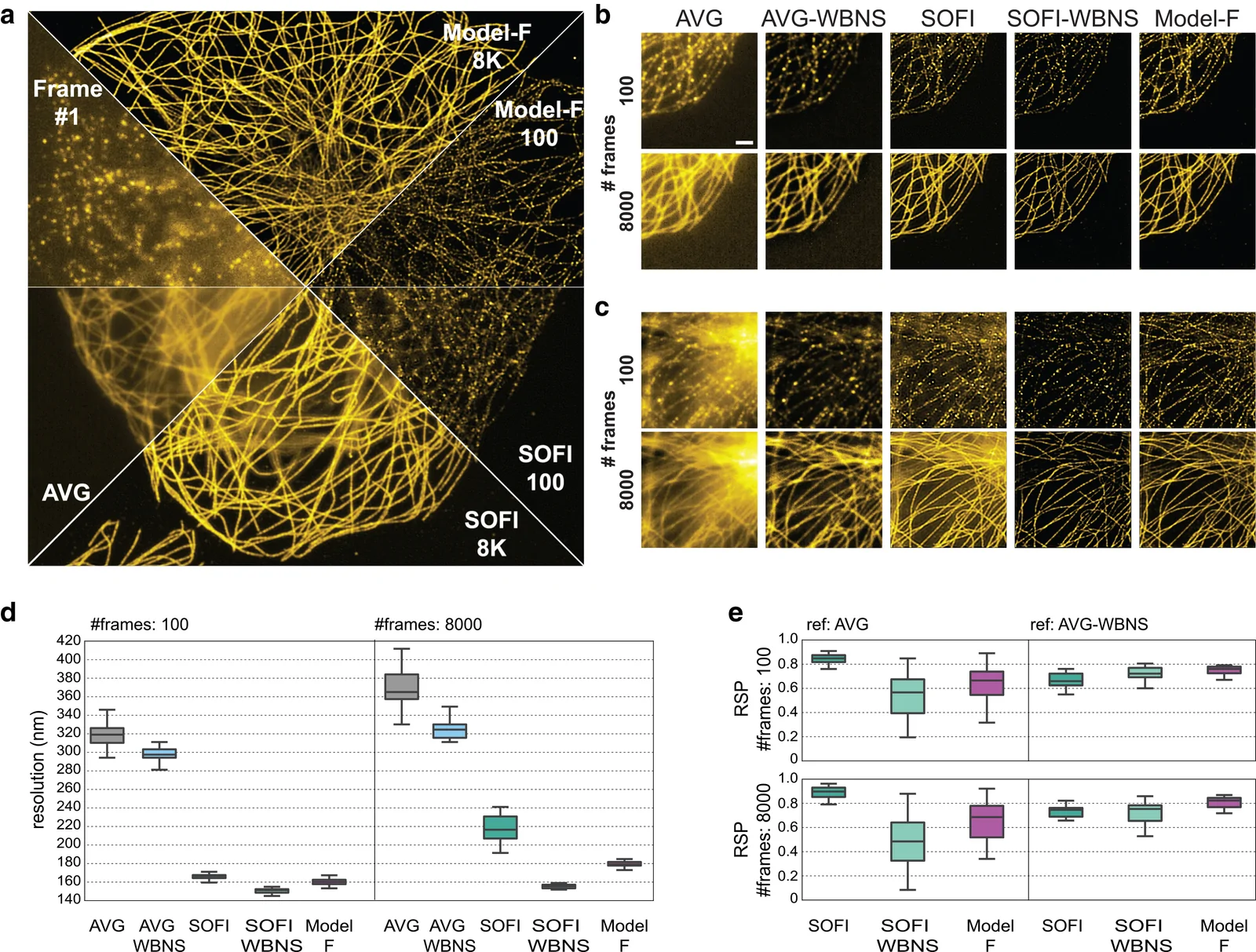
Generalization of RESURF to slow-blinking samples tested by a public SOFI data set expressing microtubules and labeled by self-blinking dyes.^49^ (a–c) COS-7 cell, immunostained for microtubules with Abberior FLIP 565. Qualitative comparison of SR reconstructions using 100 or 8,000 frames by our models, SOFI, and eSRRF. Small regions of 128 × 128 pixels are cropped from a high-structural-density (c) and a low-structural-density (b) area. (d and e) Quantitative comparisons via resolution estimation and resolution-scaled Pearson correlation (RSP). SOFI-8000 is the average of SOFI reconstructions using 500 frames. Model-F for 100 and 8,000 frames is calculated by averaging the model reconstructions used by 20 frames as input. Scale bars: 2μm. Each boxplot depicts the median value (center line), 25th and 75th percentiles (box edges), and 1.5 IQR (interquartile range) (whiskers).

**Figure 6:**
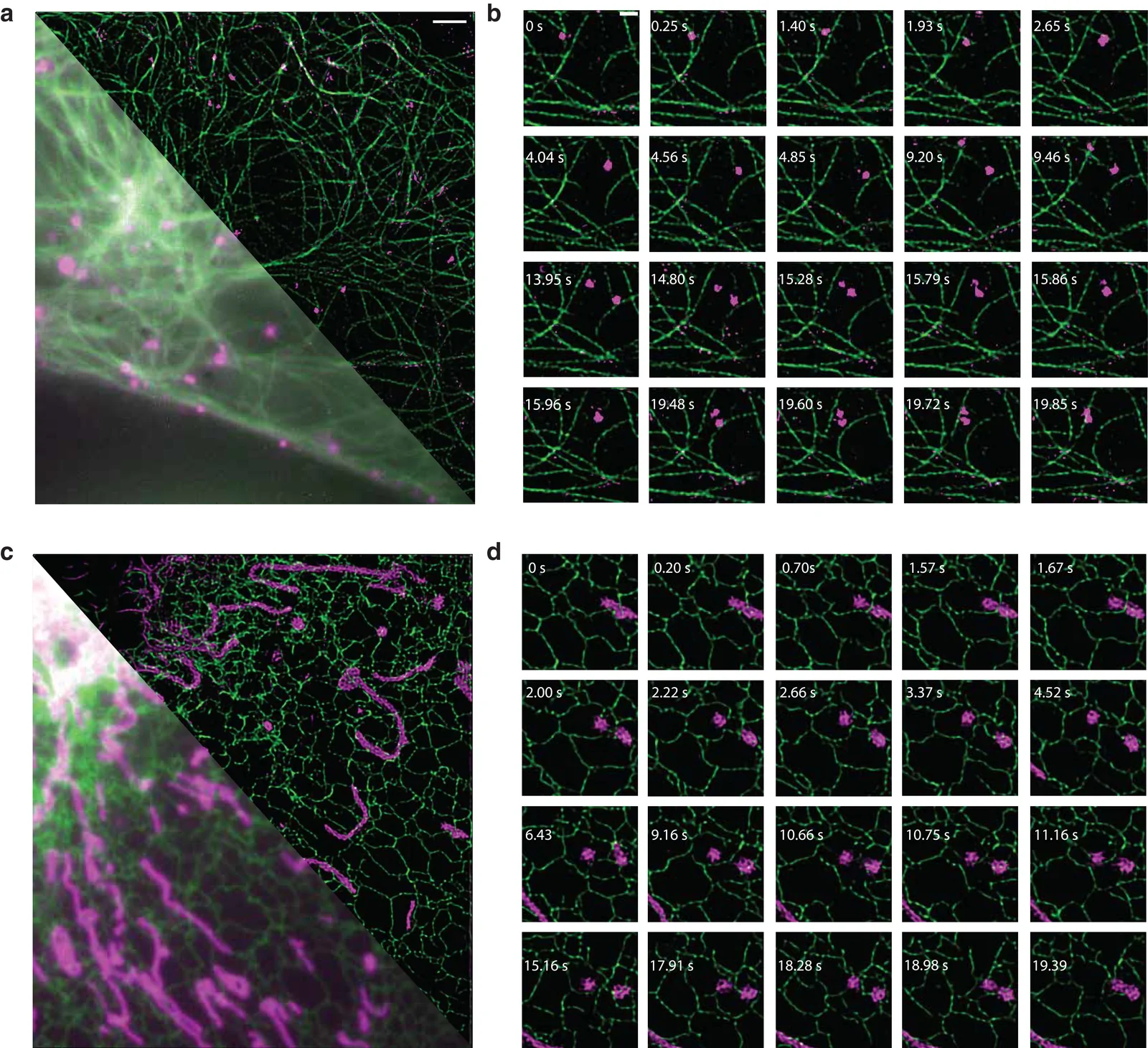
RESURF observation of dynamic biological processes in public data set.^47^ (a) Two-color image of microtubules (green) and vesicles (magenta) in Beas2B cells. (b) Example of vesicle transportation dynamics. (c) Two-color image of endoplasmic reticulum (ER; green) and mitochondria (magenta) in Beas2B cells. (d) Example of mitochondrial fission at ER-mito contact site. Wide-field images are averaged projections of the first 8 frames, and RESURF results are reconstructed by rolling an 8-frame window and using Model-F. Time labels belong to the first frame in each window. Scale bars: 5 μm (a) and 1 μm (b).

We further investigated the generalization of models trained with synthetic data to different microscopes using public data sets. As we did not vary the PSF width in our simulations, our models require retraining to perform well in very different optical configurations (see section transfer learning with small experimental data set). Therefore, we only selected data sets with an FWHM close to our synthetic images (between 2.5 and 3.2 pixels). First, we tested a data set that uses self-blinking dyes to label microtubule structures with our model trained for live-cell conditions.^49^ This public data set was aimed at higher orders of SOFI analysis, using statistics from many frames with slower and more sparse blinking conditions than the data sets we tested in previous sections. As there are not enough emitters activated over 20 frames to reconstruct a complete structure, we averaged consecutive model predictions. By using either 100 or 8,000 frames in total (by averaging 5 and 400 SR predictions), we compared them with the corresponding SOFI images. The conditions of the experiment were ideal for SOFI, and both model and SOFI reconstructions showed comparable results (Figure 5). Next, we used two data sets originally acquired for the deep-learning-based SR technique called SFSRM.^47^^,^^50^ SFSRM used single frames to reach SMLM-level resolutions. While the method can reach high temporal resolution, as stated in the study, this is an ill-posed problem and comes with strict limitations. Especially at low SNR levels, the method requires denoising prior to inference, making its application more complicated under live-cell conditions. The data set includes two highly dynamic intracellular structures labeled by fluorescent proteins, requiring high temporal information. Therefore, we used an 8-frame model, and we did not apply any pre-processing before the model inference. We reconstructed an SR movie of microtubule-vesicle interactions during vesicle transport and mitochondrial fission at the endoplasmic reticulum contact site (Figure 6). We showed that the RESURF model can be used for a wide range of SNR, background, and blinking conditions and is applicable to data sets from different microscopes with similar optics without retraining.

### Transfer learning with small experimental data set

The previous models are trained only with simulations and focused on low- and ultra-low-SNR conditions with a narrow PSF FWHM (around 2.7 pixels). We retrained the network to achieve reliable SR prediction for high-SNR DNA-PAINT experiments with a significantly larger PSF (FWHM of 3.8 pixels). We used transfer learning for the new model and trained it with experimental microtubule data acquired from fixed-cell samples. Therefore, we were able to evaluate the model’s performance in high-SNR applications and examine its consistency compared to models trained with simulations only. DNA-PAINT labeling can be used to tailor fluorescent blinking to achieve both SMLM and SOFI imaging.^45^ As a test case, we chose a fast-blinking condition, and as a result of abundant exchangeable bright emitters inside the DNA-PAINT buffer, it allows long-term high-SNR data acquisition. We showed that RESURF speeds up both data acquisition and computation times, making it suitable for real-time high-throughput imaging.

**Figure 7:**
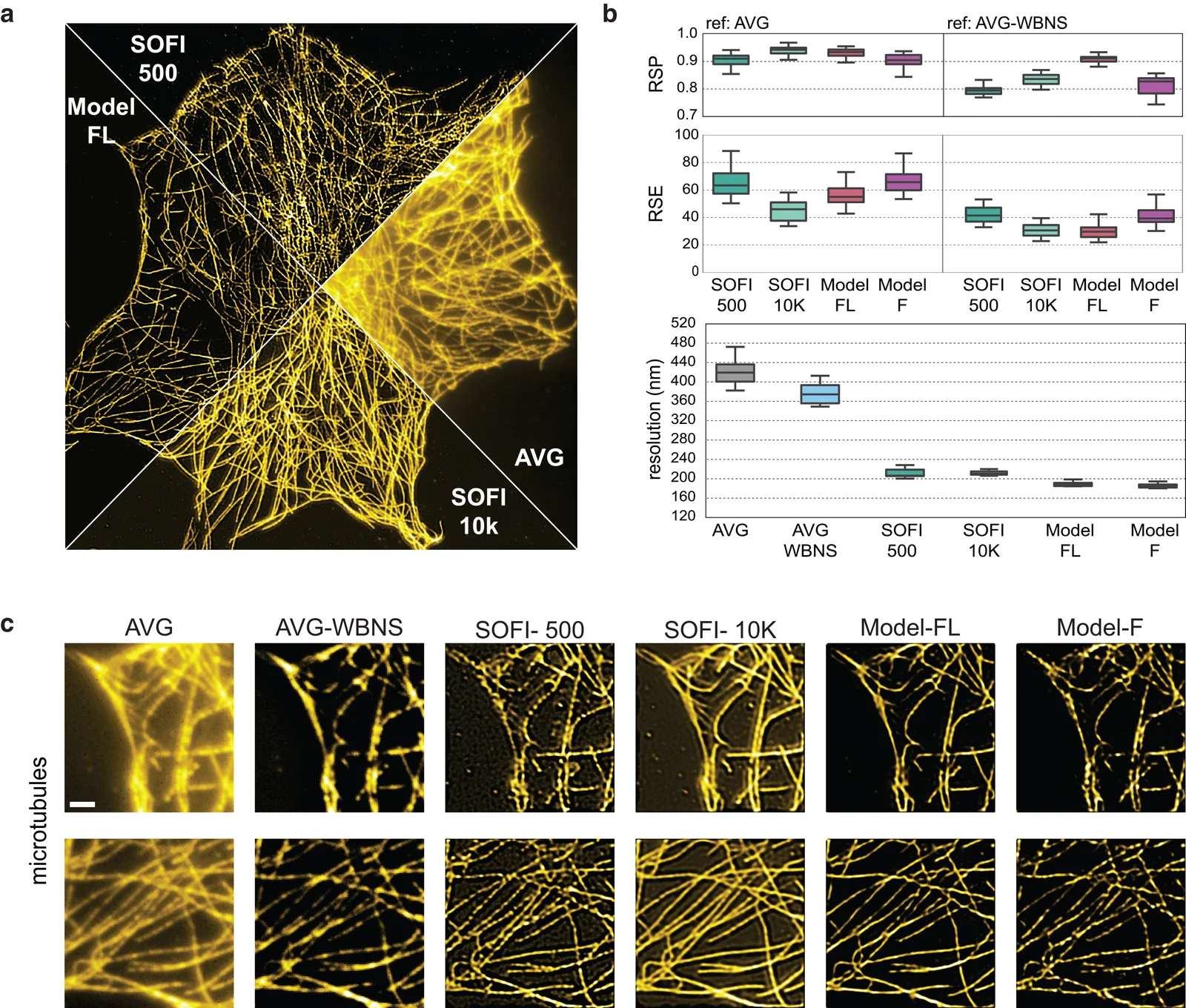
RESURF fine-tuned by small experimental data set enables high-throughput SR imaging for fast-blinking DNA-PAINT samples. (a) SR reconstructions and averaged wide-field image of microtubules in a COS7 cell, labeled by DNA-PAINT. (b) Corresponding evaluation scores of resolution estimation and resolution-scaled Pearson correlation (RSP) for two different reference images (average [AVG] and post-processed average [AVG-WBNS] of diffraction-limited frames). (c) Qualitative comparison of SR reconstructions by our models and SOFI with 500 and 10,000 frames for a cropped region of 128 × 128 pixels in wide field. Averaged wide-field and model predictions are reconstructed using 20 frames. Scale bar: 2μm. Each boxplot depicts the median value (center line), 25th and 75th percentiles (box edges), and 1.5 IQR (interquartile range) (whiskers).

Using pre-trained weights from synthetic data allows us to leverage learned features, speeding up the training process and improving generalization, which is especially useful when constrained by limited microscopy data.^26^^,^^51^^,^^52^ We trained the model using a data set of only 500 patches of 128 × 128 pixels. We created GT SOFI images by using 500 frames and trained the models using 20 frames as input. As shown in Figure S4 and for live-cell experiments, limiting the number of frames used for target SOFI reconstruction is a crucial step to prevent hallucinations and encourage generalization. Intuitively, using more frames for SOFI could lead to better target images for training. However, it also brings a risk of hallucinations if the input images do not have the same emitters activated during 20 frames. This can encourage the model to memorize the patterns instead of extracting temporal correlations. Therefore, although our data set contains 10,000 frames of acquisition, we limit the target SOFI reconstructions to 500 frames to reduce the risk of hallucinations. The results are presented in Figure 7, and the model predictions are compared to SOFI with both 500 and 10,000 frames. Similar to the simulations, the models effectively approximate the filaments by doubling the resolution and show high RSP scores. This proves that RESURF is suitable for retraining with small data sets to learn different microscopy settings, allowing easy adaptation and fast training for different experimental conditions.

## Discussion

We offer an effective, lightweight deep learning solution for ultrafast real-time super-resolution fluctuation imaging (RESURF). To utilize temporal information and reach a high computational speed, we adopted a deep learning architecture with an RNN called MISRGRU. We designed RESURF to enhance both spatial and temporal resolution while remaining adaptable to a range of imaging conditions. By the inherent ability of recurrent networks to process sequential data, our method could serve as a real-time alternative to any fluctuation-based SR imaging method. We also provide our simulation tool and data set as a benchmarking platform for fluctuation-based SR techniques, which can be used for both training and testing. We optimized our method with synthetic and experimental data sets to reconstruct second-order SOFI images with approximately 400-fold acceleration in processing time compared to conventional SOFI and using only 8–20 frames. RESURF allows forecasting of SR images from raw data during the ongoing experiment and reduces background artifacts while doubling the spatial resolution. We note that the latency of models benchmarked with a single GPU (NVIDIA RTX A5000) after TensorRT optimization could be further improved with more investment in hardware and parallelization. Although reducing the number of input frames enhances temporal resolution and increases the computational speed, this alone does not guarantee real-time deployment. Additional demands, such as pre-processing steps and model complexity, which make other methods unsuitable,^23^^,^^47^ are equally critical for a computationally manageable training pipeline and achieving real-time performance. Our focus was on developing an efficient, lightweight deep learning pipeline that enables practical and accessible deployment. This has become increasingly important, as AI is progressively integrated into biomedical applications. Our models offer substantial reductions in computational cost, an important consideration to make deep learning accessible and reduce the carbon footprint.

Our model leverages temporal information to effectively capture correlated fluorophore blinking events, making it suitable for denser fluorophore labeling compared to SMLM. We observed that our approach achieves a favorable balance between spatial resolution, structural fidelity, and temporal performance. In principle, higher-resolution improvement is also possible by using higher-order SOFI images as a target. Under extremely low-SNR conditions, RESURF outperforms SOFI and eSRRF, suggesting its potential for live-cell imaging with reduced laser intensity, thereby minimizing photodamage and allowing for longer imaging sessions. In highly dynamic imaging scenarios, using a smaller number of input frames, in this case 8, reduces motion blur and enables the reconstruction of high-quality, high-frame-rate movies. Its real-time reconstruction performance allows on-the-fly segmentation, classification, or rare-event detection in super-resolved images.

We first pre-trained the model only on synthetic data sets to create foundation models. This offers a great opportunity for live-cell experiments, where collecting training data can be very challenging due to poor SNR conditions and the dynamic behavior of the sample. Next, we fine-tuned foundation models using a small amount of data, either from simulations or experiments. Leveraging pre-trained weights from synthetic data enabled the model to retain useful feature representations, thereby accelerating convergence and enhancing generalization. This is particularly valuable when only limited experimental microscopy data are available. Moreover, the models were able to generalize to different intracellular structures, including mitochondria and actin, as well as vesicles and endoplasmic reticulum acquired from a different microscopy system, as tested using public data sets. By optimizing the simulation conditions, SOFI target images resemble an incompletely sampled structure and do not have the full underlying GT.^22^ Thus, our models can extract temporal correlation between input frames without structural bias.

For our foundational model, we focused on low- and ultra-low-SNR conditions. Furthermore, we preserved the original 16-bit dynamic range of the images and did not apply min-max normalization. This design choice was intended to maintain consistent brightness relationships between independent inputs during inference. However, this increases the model’s sensitivity to high-intensity inputs, which may result in saturated predictions. Mitigation is straightforward through transfer learning using a small data set, here demonstrated for a high-SNR DNA-PAINT sample.

We optimized our training to eliminate background artifacts by post-processing target SOFI images (synthetic data set) or using longer image sequences to reconstruct the SOFI image (experimental data set). To further improve the robustness of the model, especially under challenging imaging conditions such as those encountered in live-cell cytosolic environments, several strategies can be pursued. One promising direction is enhancing the quality of target images used during training through advanced post-processing techniques, such as improved linearization, and through sophisticated pre-processing methods. These improvements are critical only for ultra-low-SNR regimes, where SOFI reconstructions tend to degrade in quality. Heterogeneous experimental conditions constitute another challenge for deep learning approaches. Multiple models could be trained for different background and SNR conditions instead of one global model and applied for different regions of the images. This approach has recently been successfully implemented for SMLM.^53^ An alternative could be increasing the number of learnable parameters by increasing the number of channels and using complementary targets, instead of one SOFI image, to balance missing structures and background artifacts. This could allow the model to learn more complex tasks. However, optimizing a larger model comes at the cost of higher computational latency and training complexity.

In summary, we present a lightweight RNN-based architecture tailored for real-time super-resolution fluctuation imaging (RESURF) within the microscopy domain. The proposed model is capable of reconstructing high-quality SR images from as few as eight input frames while minimizing background artifacts and achieving a 2-fold spatial resolution enhancement near the theoretical limit. By leveraging pre-trained weights from synthetic data sets and fine-tuning with either synthetic or fixed-cell microscopy data, the network is well suited for live-cell imaging scenarios. Thanks to its low latency and practical training workflow, the model holds strong potential for real-time sample screening and for enhancing spatio-temporal resolution during live experiments. Furthermore, fast SR streaming enables high-throughput imaging and opportunities for smart microscopy.

## Data and code availability

The synthetic and experimental data sets used in this study are publicly available at 4TU.ResearchData, while the code for simulations is available on GitHub as BlinkingSimulator. The code for training and testing the models is available on GitHub as RESURF MISRGRU.

## Acknowledgments

We thank the Grussmayer lab and the TU Delft Department of Bionanoscience for support and the members of BIOLab for their advice on network design and evaluation. We thank Dr. Daan Brinks for his feedback on the manuscript. K.S.G, M.T. and N.T. were supported by the TU Delft AI lab initiative. K.G. was supported by an ERC grant (QScope, 101165129) funded by the European Union. Views and opinions expressed are, however, those of the authors only and do not necessarily reflect those of the European Union or the European Research Council Executive Agency. Neither the European Union nor the granting authority can be held responsible for them.

## Author contributions

M.T., J.K., and K.G. conceived the idea. M.T. designed the simulations. M.T. and J.K. designed the evaluation protocol with input from K.G. and N.T. J.K. tested the MISGRU network and optimized it to create foundation models. M.T. performed further analysis to evaluate the performance and improved the simulations and network training for live-cell experiments and generalization of the models. M.T. and J.K. trained the model for transfer learning with experimental data. M.T. analyzed public data sets to test generalization. H.V. designed and performed the live-cell experiments. K.Z. and R.H. designed and performed the DNA-PAINT experiments. M.T. wrote the manuscript. K.G., N.T., and P.D. reviewed the manuscript. N.T. supervised the deep network implementation and evaluation. K.G. supervised the microscopy experiments, data analysis, and network evaluation.

## Declaration of interests

The authors declare that there are no conflicts of interest related to this article.

## Supplemental information

Document S1. Figures S1–S6

Document S2. Article plus supplemental information
