# Supplementary material for: A lightweight deep learning framework for fast, real-time super-resolution fluctuation imaging
Source: Biophys Rep (N Y). 2026 Aug 6;6(3):100279. doi: 10.1016/j.bpr.2026.100279 (PMC13524874; doi:10.1016/j.bpr.2026.100279)
Supplement: Supplementary file 1 [file mmc1.pdf]

**Biophysical Reports, Volume *6***

## Supplemental information

# A lightweight deep learning framework for fast, real-time super-resolution fluctuation imaging

**Miyase Tekpınar, Jelle Komen, Hana Valenta, Ran Huo, Klarinda de Zwaan, Peter Dedecker, Nergis Tomen, and Kristin Grußmayer**

# Supplementary Information for

# A Lightweight Deep Learning Framework for Fast, Real-Time Super-Resolution Fluctuation Imaging

Miyase Tekpınar1, Jelle Komen1, Hana Valenta3, Ran Huo1, Klarinda de Zwaan1,

, Peter Dedecker3, Nergis Tomen2, Kristin Grußmayer1*

1*Department of Bionanoscience and Kavli Institute of Nanoscience, Delft University of Technology, Delft, Netherlands.

2Department of Intelligent Systems, Delft University of Technology, Delft, Netherlands.

3 Department of Chemistry, KU Leuven, Leuven, Belgium.

*Corresponding author(s). E-mail(s): k.s.grussmayer@tudelft.nl;

Contributing authors: m.tekpinar@tudelft.nl; j.j.m.komen@tudelft.nl; r.huo@tudelft.nl; b.k.d.zwaan@tue.nl; hana.valenta@kuleuven.be; peter.dedecker@kuleuven.be; n.tomen@tudelft.nl

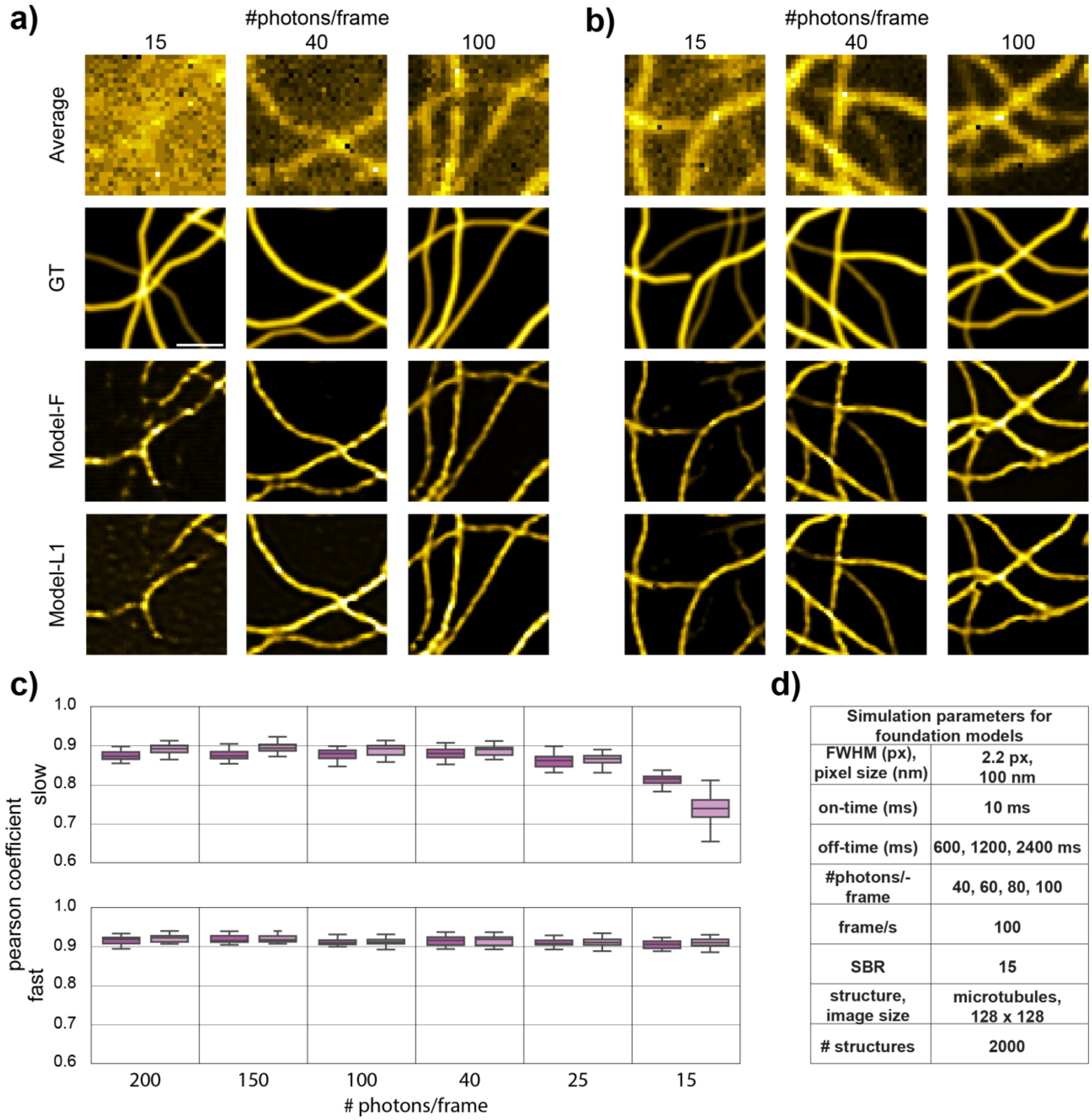


| Simulation parameters for foundation models | |
|---|---|
| FWHM (px), pixel size (nm) | 2.2 px, 100 nm |
| on-time (ms) | 10 ms |
| off-time (ms) | 600, 1200, 2400 ms |
| #photons/-frame | 40, 60, 80, 100 |
| frame/s | 100 |
| SBR | 15 |
| structure, image size | microtubules, 128 x 128 |
| # structures | 2000 |

**Supplementary Figure 1. Performance of foundation models for different SNR levels.** Model predictions and corresponding averaged wide-field and ground-truth (GT) images for **a.** slow-blinking (on: 10 ms, off: 2400 ms) and **b.** fast-blinking (on: 10ms, off: 600 ms) conditions. In each column, different SNR conditions are demonstrated. **c.** RSP scores for different SNR levels. The two box plots in each SNR level corresponds to Model-F (model with Fourier loss) and Model-L1 (model with L1 loss) trained by 20 input frames, respectively. Training set is simulated by using photon numbers of 40, 60, 80 and 100. In addition to training settings, we also tested the SNR levels below and above this range. We see that both models show high RSP scores among different SNR levels except for the lowest SNR of slow blinking images. Images simulated by 15 photons per frame represents very extreme conditions and slow-blinking conditions results in lower SNR than fast-blinking conditions as the number of active emitter per

frame is lower. Therefore in this extreme case, models show more missing structures and background. **d.** Simulation parameters for the training set used for foundation models. Scale bar: 1000 nm, all images are scaled between minimum and maximum values.

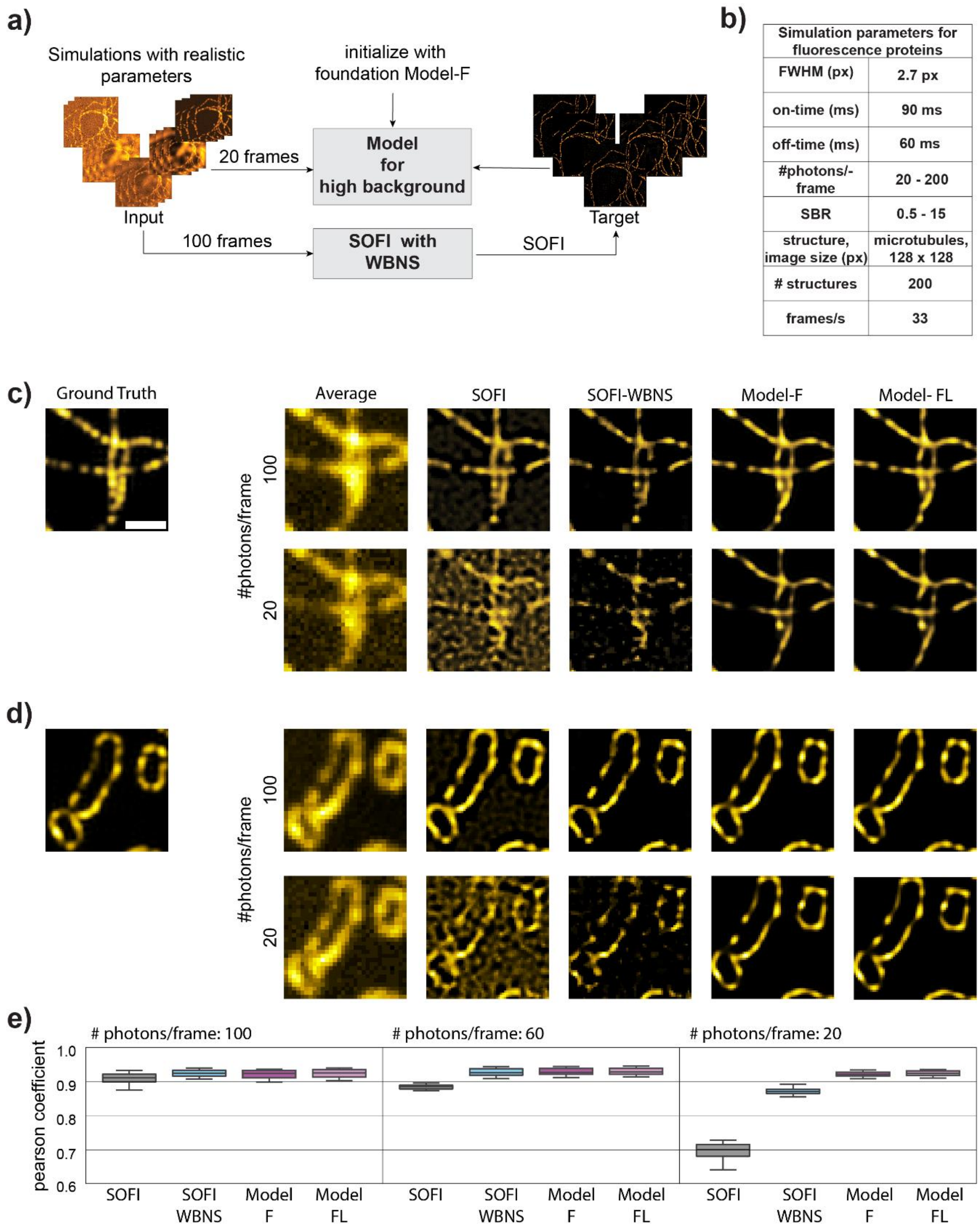


**Supplementary Figure 2. Retraining the foundation model for high background conditions and performance evaluation. a.** Training pipeline for high background conditions. We created

a new small training set for retraining. This dataset includes very high- background conditions and blinking characteristics of the fluorescence protein called ffDronpa [1]. As high-background in wide-field images resulted in higher background in SOFI targets, we used a post-processing technique called WBNS to subtract the background [2] and used those images as our target during training. **b.** Simulation parameters used to create high-background training set. The background levels are up to 60 fold higher than used to train foundation models. And all the other parameters are tailored to match to live-cell experiments with fluorescence proteins. **c-d.** Microtubules and hollow mitochondria structures under high background (SBR:0.9) and with two different SNR conditions (#photons/frame). While WBNS can eliminate the background under higher-SNR conditions, it causes more missing structures if SNR is lower. As shown in Supplementary Figure 1, in extreme SNR conditions, L1 loss performed worse than Fourier loss. Therefore during retraining, we created two models: Model-FL trained by combination of two loss functions and Model-F trained by Fourier loss. Both Model-F and Model-FL performs similarly under the tested conditions, outperforming SOFI-WBNS by reconstruction more complete structures under low-SNR. Even-though we trained with SOFI-WBNS target images, we included different SNR conditions and network could learn to both eliminate background while preserving more signal under extreme SNR conditions. **e.** Resolution-scaled Pearson correlation coefficient (RSP) scores for very low SBR (0.9) and different SNR conditions. Scale bar: 1 μm.

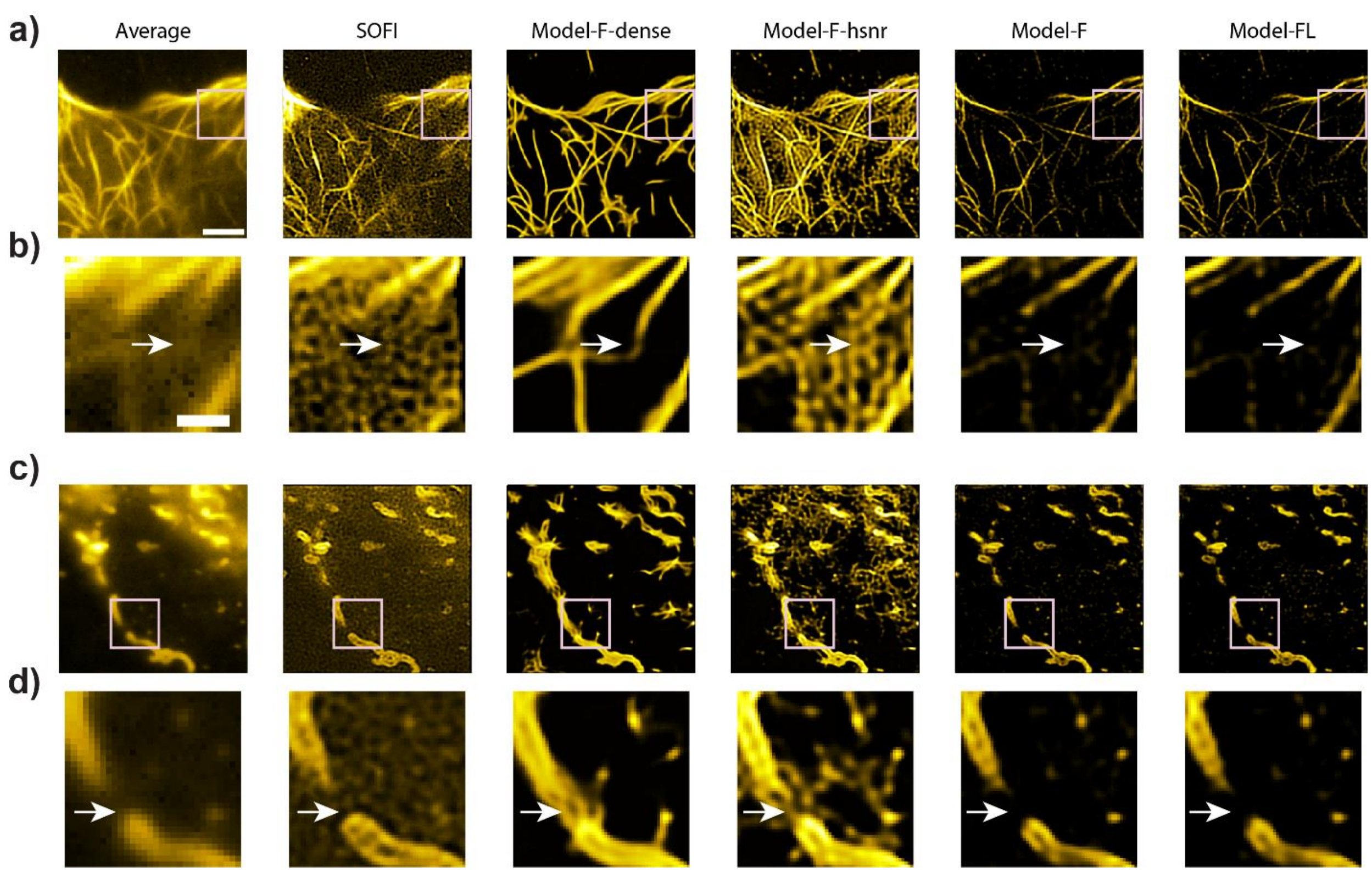


**Supplementary Figure 3. Model trained using high emitter density or very high SNR targets cannot generalize and causes hallucinations. a-c.** Comparison of SOFI and predictions of different models trained for high background data for live-cell microtubules and mitochondria

data. **b-c.** a closer look at the small regions framed in a square. **Model-F-dense** is trained by using combination of simulations with different emitter densities including a very high setting (1000-10000 emitters/$\mu m^2$). Model-F-dense can differentiate the pixels on microtubules regions from background very well resulting in a very high contrast image. However including high emitter densities results in underrepresentation of sparse random emitters and encourages the network to memorize filamentous structures. In the close look at b, we see that model connects the pixels with higher signal than the background. In addition, this also affects that model cannot recover the patterns which is not trained on, such as mitochondria (c-d). And similarly, connect the pixels with high signal together to create filamentous like structures. **Model-F-hsnr** is trained by using a SOFI target reconstructed by high SNR versions of diffraction limited image sequences. For this model, we created 1000-frames of high SNR movie to create a SOFI target and degraded the first 20-frames by changing SNR and background levels during training while still keeping the target image same. This means that the emitters which are not activated during 20-frames, could still appear in the SOFI reconstruction using 1000 frames. As a result, the Model-F-hsnr becomes very sensitive to background noise and hallucinates some very bright structures in the background. Model-F and Model-FL are the same model used in main text, in Figures 3,4,6,7. Both model trained by using structures simulated by using 1000 emitters/ $\mu m^2$ , and 100-frames SOFI reconstruction are used as a target image during training. By limiting the emitter density and optimizing the target image quality, we achieved a training strategy to prevent hallucinations and to generalize to different structures. Scale bars: 3 μm (a), 1 μm (b)

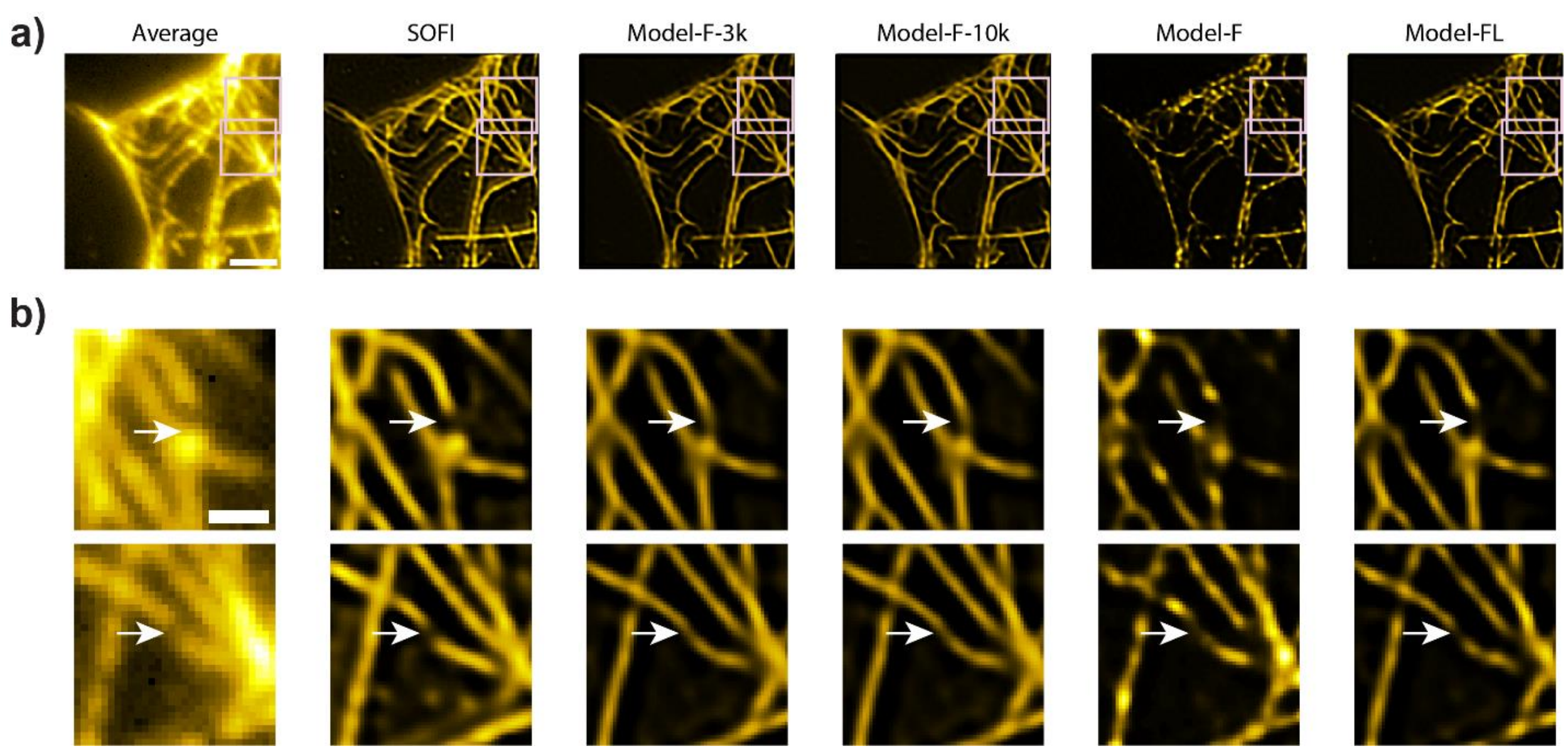


**Supplementary Figure 4. Optimizing the number of frames to reconstruct SOFI images (target) while retraining with experimental data. a-b.** Comparison of SOFI and predictions of different models retrained by experimental DNA-PAINT dataset and a closer look at the areas in square regions. **Model-F-3k** and **Model-F-10k** is trained by using SOFI targets reconstructed with 3,000 and 10,000 frames. In both models, we see that models are biased to create

filamentous like structures by connecting small gaps. On the other hand, **Model-F** and **Model-FL** achieved not to hallucinate and recovered very low signal in the blank areas similar to SOFI and averaged wide-field images. They are the same models used in Figure 5 in the main text and they are trained by using the SOFI images reconstructed by using 500 frames. We also tried to train another model by using 100 frames SOFI targets however training was failed as the SOFI reconstruction had very low SNR. Scale bars: 3 µm (a), 1 µm (b)

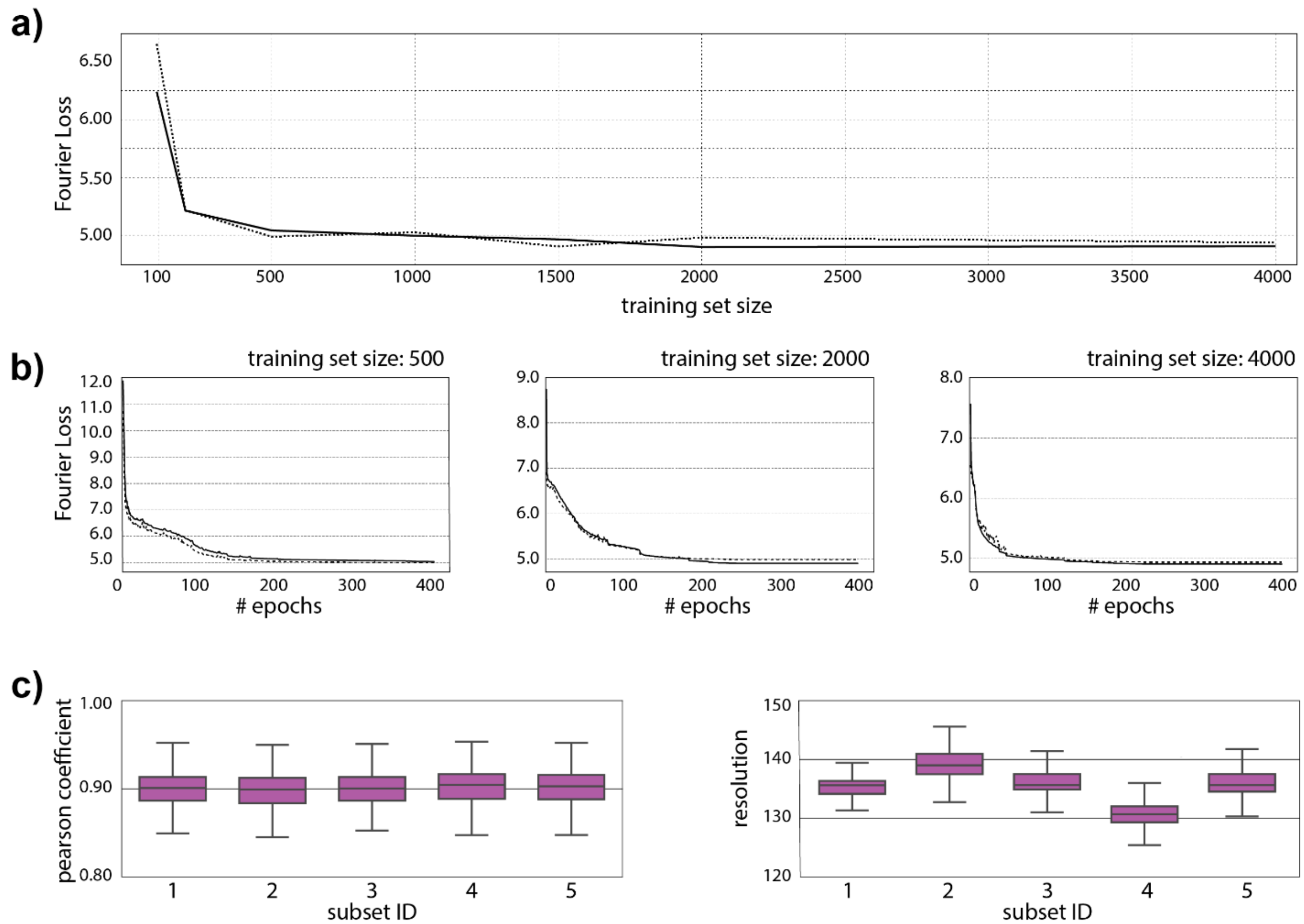


**Supplementary Figure 5. Dependency of training performance on training dataset. a.** Training and validation error of the model after 400 epochs for varying training set size. It converges quickly around the training set size of 2000, showing that the model does not require large datasets. **b.** Individual learning curves for the models trained by using dataset size of 500, 2000 and 4000. The larger datasets converged faster than smaller datasets. **c.** K-fold cross-validation analysis. We split the dataset in 5-folds and used each fold as validation once and the rest as training set to create 5 independent models. All models showed similar scores in resolution and RSP analysis indicating that the models have high confidence and are not affected by the variations in the training dataset.

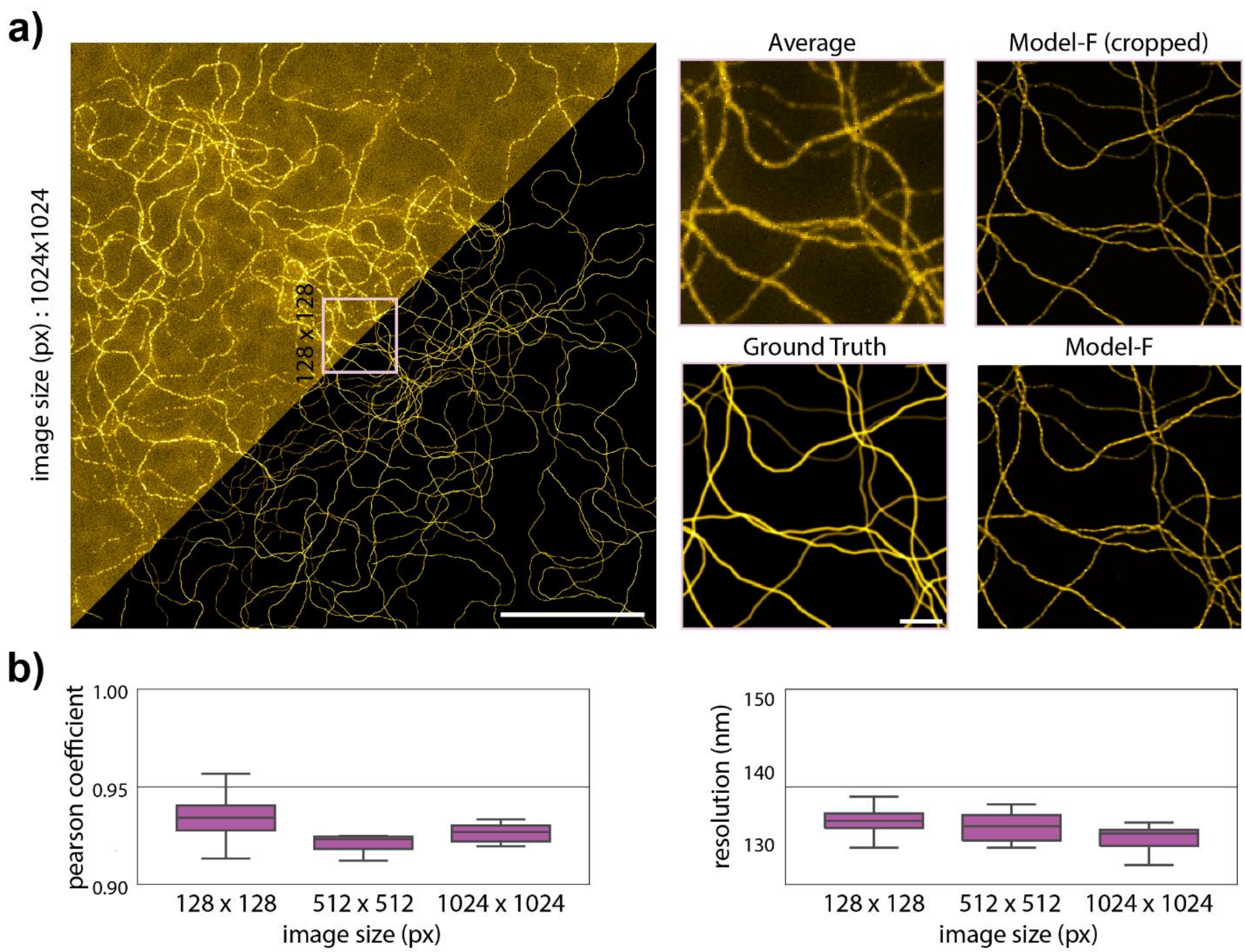


**Supplementary Figure 6. a.** Model prediction of an input region of 1024x1024 pixels, and close look at the 128x128 pixels central area inside the square region**. b.** RSP and resolution scores of model prediction for input images of different sizes. Model-F(cropped) is the cropped region from the model prediction for the image size of 1024x1024. And Model-F is the prediction of the model by using wide-field central region of 128x128 as an input. We could not detect any significant difference between two images. We also evaluated the model performances for different input image sizes and showed that model perform similar for larger images even though it is trained only using images of 128x128 pixels. Scale bars: 25 and 2 µm

[1] “Fast and Reversible Photoswitching of the Fluorescent Protein Dronpa as Evidenced by Fluorescence Correlation Spectroscopy - ScienceDirect.” Accessed: Dec. 15, 2025. [Online]. Available: https://www-sciencedirect-com.tudelft.idm.oclc.org/science/article/pii/S0006349506718707

[2] M. Hüpfel, A. Y. Kobitski, W. Zhang, and G. U. Nienhaus, “Wavelet-based background and noise subtraction for fluorescence microscopy images,” *Biomed. Opt. Express*, vol. 12, no. 2, pp. 969–980, Feb. 2021, doi: 10.1364/BOE.413181.
